# Nanoplankton: The dominant vector for carbon export across the Atlantic Southern Ocean in spring

**DOI:** 10.1126/sciadv.adi3059

**Published:** 2023-12-01

**Authors:** Raquel F. Flynn, Lumi Haraguchi, Jeff McQuaid, Jessica M. Burger, Percy Mutseka Lunga, Luca Stirnimann, Saumik Samanta, Alakendra N. Roychoudhury, Sarah E. Fawcett

**Affiliations:** ^1^ Department of Oceanography, University of Cape Town, Cape Town, South Africa.; ^2^ Finnish Environment Institute (SYKE), Helsinki, Finland.; ^3^ Integrative Oceanography Division, Scripps Institution of Oceanography, La Jolla, CA, USA.; ^4^ Department of Genetics, University of Pretoria, Pretoria, South Africa.; ^5^ Department of Earth Sciences, Stellenbosch University, Stellenbosch, South Africa.; ^6^ Marine and Antarctic Research Centre for Innovation and Sustainability (MARIS), University of Cape Town, Cape Town, South Africa.

## Abstract

Across the Southern Ocean, large (≥20 μm) diatoms are generally assumed to be the primary vector for carbon export, although this assumption derives mainly from summertime observations. Here, we investigated carbon production and export potential during the Atlantic Southern Ocean’s spring bloom from size-fractionated measurements of net primary production (NPP), nitrogen (nitrate, ammonium, urea) and iron (labile inorganic iron, organically complexed iron) uptake, and a high-resolution characterization of phytoplankton community composition. The nanoplankton-sized (2.7 to 20 μm) diatom, *Chaetoceros* spp., dominated the biomass, NPP, and nitrate uptake across the basin (40°S to 56°S), which we attribute to their low iron requirement, rapid response to increased light, and ability to escape grazing when aggregated into chains. We estimate that the spring *Chaetoceros* bloom accounted for >25% of annual export production across the Atlantic Southern Ocean, a finding consistent with recent observations from other regions highlighting the central role of the phytoplankton “middle class” in carbon export.

## Teaser

In spring, short-lived blooms of medium-sized diatoms drive elevated carbon production and export across the Southern Ocean.

## INTRODUCTION

In the open Southern Ocean, primary production is strongly iron-limited (*1*, *2*). Phytoplankton require iron for various photosynthetic and metabolic processes, with the assimilation of nitrate [NO_3_^−^; the most oxidized form of bioavailable nitrogen (N)] requiring far more iron than the consumption of regenerated N [e.g., ammonium (NH_4_^+^) and urea] (*3*). Phytoplankton that rely on NO_3_^−^ as their primary N source (e.g., diatoms) (*4*) are thus more sensitive to iron limitation than phytoplankton that mainly assimilate regenerated NH_4_^+^ (*3*). In the framework of the “new production paradigm,” phytoplankton growth supported by new N (e.g., NO_3_^−^ supplied during deep winter mixing) must be balanced on an annual basis by the flux of organic matter out of the mixed layer (i.e., export production) (*5*, *6*). Since the low-iron state of the upper Southern Ocean constrains the extent to which upwelled NO_3_^−^ can be consumed by phytoplankton, it also limits biological carbon export (*1*, *7*).

For over four decades, ecological theory has suggested that across the global ocean, large phytoplankton (typically diatoms >20 μm) proliferate under nutrient-replete conditions, an idea based on the assumption that maximum nutrient uptake rates generally increase with cell volume (*8*). These large phytoplankton are responsible for high rates of primary and new production, with their eventual senescence causing large sinking fluxes that drive elevated carbon export (*6*). In contrast, small cells are typically associated with nutrient-deplete conditions, low rates of productivity, and enhanced nutrient recycling (i.e., the microbial loop) (*9*, *10*). They also contribute little to the sinking flux and, by extension, carbon export (*11*, *12*). This theoretical framework has generally been accepted, and to some extent observed, for the open Southern Ocean (*13*, *14*). However, recent studies from other regions have shown that the phytoplankton “middle class” (i.e., the nanoplankton; ~3 to 20 μm) is central to primary productivity and carbon export, even in high-nutrient waters; the idea that nanoplankton predominantly contribute to the microbial loop thus appears to be an oversimplification (*15*–*18*). These recent studies suggest that top-down factors (e.g., zooplankton grazing) exert the major control on nanoplankton production and carbon export, both directly via grazing and indirectly by driving changes in nutrient availability (e.g., N recycling) and phytoplankton community composition (*15*, *16*).

The surface waters of the open Southern Ocean are replenished with iron during deep winter mixing, with additional (smaller) inputs via diapycnal diffusion and Ekman upwelling that occur year-round (*19*–*21*). In spring, phytoplankton are released from light limitation as daylight hours increase and the mixed layer shoals; along with the iron supplied during winter, the elevated light favors bloom initiation as phytoplankton begin to consume the available macronutrients (i.e., NO_3_^−^, phosphate, and silicate) (*1*, *22*). Consistent with the ecological theory outlined above, large diatoms are generally thought to dominate the open Southern Ocean spring bloom as they can achieve high maximum nutrient uptake rates and are resistant to grazing by microzooplankton (*8*, *23*, *24*, *25*, *26*). These diatoms are also considered the major vectors for carbon export because of their biogenic silica (bSi) frustules, which cause them to sink rapidly out of the mixed layer, increase their resistance to bacterial degradation, and ballast zooplankton fecal pellets (*24*, *25*, *27*). As the growth season progresses and iron is depleted, the Southern Ocean phytoplankton community is thought to shift toward smaller taxa (e.g., nanoflagellates) that preferentially consume regenerated N (*28*, *29*) and/or to microplankton-sized diatoms (e.g., *Fragilariopsis*) that can store large quantities of iron intracellularly (*30*, *31*). This shift decreases the potential for carbon export, both directly, as unballasted small phytoplankton sink slowly and are generally remineralized within the upper 200 m (*32*), while the large, heavily silicified diatoms appear to be inefficient carbon exporters (*26*, *27*), and indirectly, as regenerated N uptake supports no CO_2_ removal from the mixed layer in a mass balance sense (*5*, *6*).

Studies using satellite observations and autonomous float-based measurements of chlorophyll a indicate that the seasonal modulation of phytoplankton blooms in the Southern Ocean is the result of a tight coupling between bottom-up (mainly iron and light) and top-down (grazing pressure and viral lysis) processes (*33*, *34*). Recently, Arteaga *et al*. (*34*) showed that the Southern Ocean’s annual cycle of phytoplankton productivity includes a period of rapid growth in spring as light limitation is alleviated, with phytoplankton initially outpacing their grazers. The authors found that phytoplankton achieve their maximum growth rates in early spring, well before the peak in photoautotrophic biomass that characterizes the summer. We hypothesize that the spring bloom (i.e., the period of rapid phytoplankton growth) should be associated with elevated carbon export in addition to high rates of primary production, made possible by the nonlimiting iron (and in some regions, silicate) and light conditions, as well as slow zooplankton grazing. To investigate this hypothesis, we measured the rates of net primary production (NPP) and N (as NO_3_^−^, NH_4_^+^, and urea) and iron (as labile inorganic iron and organically complexed iron) uptake by three phytoplankton size classes (0.3 to 2.7 μm—picoplankton, 2.7 to 20 μm—nanoplankton, and 20 to 200 μm—microplankton) at four stations representing each of the major hydrographic zones of the open Southern Ocean [i.e., the Subantarctic Zone (SAZ), Polar Frontal Zone (PFZ), Open Antarctic Zone (OAZ), and Marginal Ice Zone (MIZ)]. We interpret the uptake rates in the context of coincident measurements (from the four experimental stations plus eight ancillary stations) of macronutrient and iron concentrations, as well as phyto- and zooplankton community composition. From the nutrient data, we also estimate net community production (NCP), which provides a measure of carbon export (*35*). We conclude by placing our springtime observations into a broader temporal context by synthesizing the observations available for the phytoplankton growth season, with the goal of understanding the biogeochemical implications of the spring bloom for the succeeding phytoplankton community.

## RESULTS

Our study was conducted in the Atlantic sector of the Southern Ocean, which experiences different physical (*36*) and chemical (*37*, *38*) conditions from the Pacific and Indian sectors. As such, while our discussion of the physicochemical conditions and their influence on phytoplankton community dynamics likely has implications for the Southern Ocean as a whole, it is focused on the Atlantic sector.

### Hydrographic and biogeochemical context

**Fig. 1.**
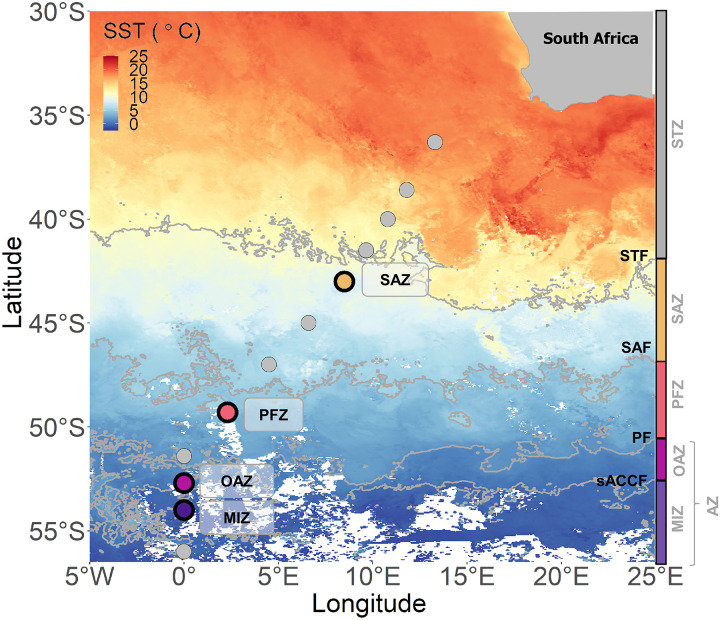
Station locations. Map of the Atlantic sector of the Southern Ocean showing the sampling stations overlaid on the average sea surface temperature (SST) recorded in November 2019 (https://oceancolor.gsfc.nasa.gov/l3/). The fronts are identified by the gray lines [Subtropical Front (STF), Subantarctic Front (SAF), Polar Front (PF), and southern boundary of the Antarctic Circumpolar Current Front (sACCF) (*40*)], and the hydrographic zones are indicated to the right of the map by the colored rectangles [gray, Subtropical Zone (STZ); yellow, Subantarctic Zone (SAZ); orange, Polar Frontal Zone (PFZ); pink, Open Antarctic Zone (OAZ); purple, Marginal Ice Zone (MIZ); together, OAZ and MIZ constitute the Antarctic Zone (AZ)]. The station positions are denoted by the circles, with the colors indicating the experimental stations and the gray circles indicating the ancillary stations.

**Fig. 2.**
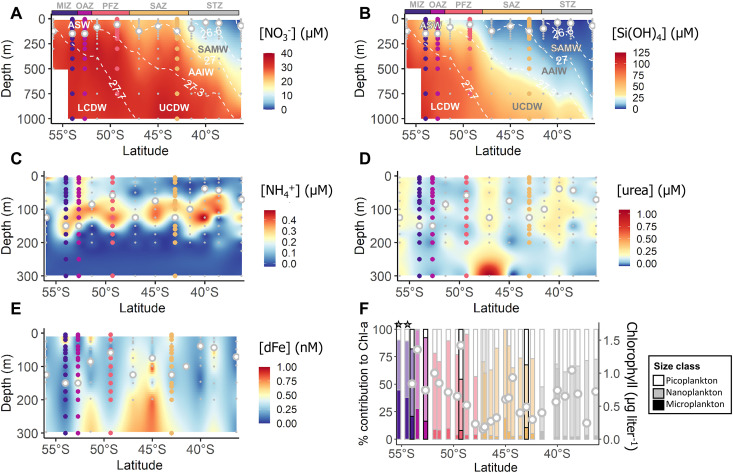
Physicochemical context. Section plots showing the concentrations of (**A**) nitrate ([NO_3_^−^]), (**B**) silicate {[Si(OH)_4_]}, (**C**) ammonium ([NH_4_^+^]), (**D**) urea-N ([urea]), and (**E**) dissolved iron ([dFe]), and (**F**) a bar plot showing total surface chlorophyll a concentration (white dots, right *y* axis) and the relative contribution of each phytoplankton size class to chlorophyll a (colors, left *y* axis) for samples collected along the Good Hope line in spring. The hydrographic zones are indicated at the top of panels (A and B) [gray, STZ; yellow, SAZ; orange, PFZ; pink, OAZ; purple, MIZ; together, OAZ and MIZ constitute the AZ]. The station positions are shown in (A) to (E), with the colored circles denoting the experimental stations and the gray circles denoting the ancillary stations. The different water masses are identified in (A) and (B) by the density contours (ASW, Antarctic Surface Water; LCDW, Lower Circumpolar Deep Water; UCDW, Upper Circumpolar Deep Water; AAIW, Antarctic Intermediate Water; SAMW, Subantarctic Mode Water), and the mixed-layer depth (MLD) is indicated on (A) to (E) by the white filled circles. In (F), the black outlined bars indicate the four experimental stations, the colored bars represent the hydrographic zones, and the shading shows the plankton size classes (white, picoplankton; opaque, nanoplankton; solid, microplankton). The stars in (F) indicate stations where the surface chlorophyll a concentrations exceeded the right *y* axis scale (3.0 μg liter^−1^ and 1.8 μg liter^−1^ at 55.5°S and 55°S, respectively).

Along a transect of the Atlantic Southern Ocean between South Africa and MIZ [the Good Hope line (*39*); 36°S to 56°S, occupied in early spring; Fig. 1] surface water density was highest at the southernmost station (56°S), decreasing northward (Fig. 2, A and B) due to warming and salinification of Antarctic Surface Waters (ASW) during equatorward Ekman transport. The Subtropical Front (STF), Subantarctic Front (SAF), Polar Front (PF), and southern Antarctic Circumpolar Current Front (sACCF), which divide the Southern Ocean into its major hydrographic zones, were located at approximately 41°S, 47°S, 52°S, and 53°S, respectively (*40*).

**Fig. 3.**
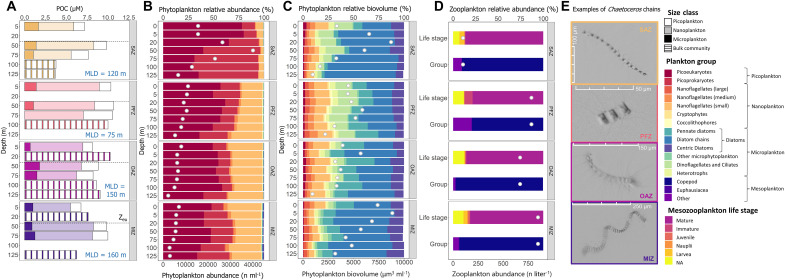
Plankton biomass and community composition. Bar plots for the four experimental stations of (**A**) size-fractionated particulate organic carbon (POC) concentration, (**B**) relative phytoplankton abundance and total cell counts between 5 and 125 m, (**C**) relative and total phytoplankton biovolume between 5 and 125 m, and (**D**) relative and total mesozooplankton counts from Bongo net collections (integrated surface to 200 m). (**E**) Images taken by the CytoSense flow cytometer of the dominant *Chaetoceros* spp. chains present at the experimental stations. The stations are labeled by zone on the right *y* axes of all the panels. The black dashed line in (A) indicates the euphotic zone depth (*Z*_eu_), MLD is annotated on the plots, and the shading of the bars denotes the different plankton size classes (white, picoplankton; opaque, nanoplankton; solid color, microplankton). At the depths where size-fractionated POC concentrations were not measured, only the bulk values are shown (striped bars). The white dots in (B) and (D) indicate the total plankton counts, and those in (C) indicate the total biovolume. The bars in (D) show the different mesozooplankton life stages and main groups identified at the experimental stations.

The mixed-layer depth (MLD) was predominantly controlled by salinity and was always deeper than the base of the euphotic zone (*Z*_eu_; table S1). The deepest mixed layer was observed in the MIZ (160 m) and the shallowest in the PFZ (75 m), while the *Z*_eu_ was similar at all stations (37.5 to 45 m). The rates of NPP and N and iron uptake were trapezoidally integrated to the MLD rather than the *Z*_eu_ given that phytoplankton biomass was elevated to at least 125 m (Fig. 3, A to C). Since we have no rate measurements from the base of the mixed layer, we set the values at this depth to 0 μM day^−1^ (*41*).

As expected, the concentrations of NO_3_^−^ and silicate [Si(OH)_4_] were lower in the mixed layer than the subsurface, and the mixed-layer concentrations decreased northward (Fig. 2, A and B). The highest mixed-layer NO_3_^−^ was observed in the OAZ and MIZ [Antarctic Zone (AZ); regional average of 27.4 ± 0.7 μM; *n* = 3 stations], while Si(OH)_4_ was highest in the MIZ (51.3 ± 14.4 μM; *n* = 2 stations). The lowest mixed-layer NO_3_^−^ and Si(OH)_4_ concentrations were both observed in the SAZ (20.5 ± 1.1 μM and 5.9 ± 0.7 μM, respectively; *n* = 3 stations). The concentrations of regenerated N (i.e., NH_4_^+^ and urea-N, with the latter hereafter referred to as urea) were low in the surface, increasing with depth to reach a maximum just below the *Z*_eu_ (Fig. 2, C and D). Mixed-layer concentrations of NH_4_^+^ were highest in the OAZ (0.26 ± 0.05 μM; *n* = 1 station) and lowest in the SAZ (0.12 ± 0.09 μM; *n* = 3 stations), while urea concentrations were highest in the MIZ (0.14 ± 0.05 μM; *n* = 2 stations) and lowest in the OAZ (0.05 ± 0.02 μM; *n* = 1 station).

Mixed-layer dissolved iron concentrations ([dFe]) were generally >0.2 nM (Fig. 2E) at all depths across the transect. The highest dFe was measured in the MIZ surface ([dFe] = 0.75 nM at 25 m), while the average mixed-layer dFe concentration was highest in the SAZ (regional average of 0.38 ± 0.1 nM; *n* = 3 stations) and lowest in the OAZ (0.26 ± 0.15 nM; *n* = 1 station).

### Phytoplankton biomass and distributions

Chlorophyll a concentrations were highest in the MIZ mixed layer (regional average of 2.7 ± 1.1 μg liter^−1^; *n* = 3 stations) and lowest in the SAZ (0.4 ± 0.2 μg liter^−1^; *n* = 3 stations), particularly near the SAF (Fig. 2F and fig. S1). Across the transect, chlorophyll a was dominated by nanoplankton, which contributed an average of 65 ± 12%. The pico- and microplankton contributed less to chlorophyll a (transect average of 25 ± 14% and 10 ± 11%, respectively), although the microplankton contribution increased in the MIZ to 34 ± 10%.

The physicochemical conditions at all stations in the same hydrographic region were generally similar, as were the phytoplankton size distributions (Fig. 2). We thus take the experimental stations as broadly representative of their respective hydrographic regions. At the experimental stations, the euphotic zone-average bulk particulate organic carbon (POC) concentrations ranged from 8.1 ± 0.8 μM in the MIZ to 6.5 ± 0.4 μM in the OAZ (Fig. 3A). Biomass was dominated by the nanoplankton, which contributed an average of 68 ± 7% to total POC across the transect. The picoplankton contributed 13 ± 6% to the total POC, while the microplankton contributed 19 ± 6%.

The mixed-layer phytoplankton community at all the experimental stations was numerically dominated by picoplankton, with picoprokaryotes being the most abundant group in the SAZ (44 ± 27% of the total phytoplankton cells counted), while picoeukaryotes dominated at the other stations (52 ± 5%) (Fig. 3B). We observed a southward shift in community composition, with nanoflagellates increasing in abundance relative to picoplankton south of the SAF (nanoflagellates contributed 6 ± 3% of the counted cells in SAZ, versus 30 ± 5% at the other stations). The highest total cell abundances were observed in the SAZ (20,054 ± 10,966 cells ml^−1^), while the highest total biovolume was derived for the MIZ. Although diatoms were the least abundant phytoplankton group (average of 0.8 ± 0.6% of the cells across the transect), they contributed most to biovolume (61 ± 9%; Fig. 3C). The MIZ diatoms were generally more heavily silicified Antarctic species (e.g., *Fragillariopsis* spp.), while those encountered further north, particularly in the SAZ where mixed-layer Si(OH)_4_ concentrations were low (5.2 ± 0.4 μM at the SAZ experimental station), were smaller, weakly silicified pennate diatoms (e.g., *Pseudo-nitzschia*).

The microzooplankton community was partially characterized by flow cytometry (i.e., all cells ≤1 mm could be visualized), which allowed for the enumeration of known mixotrophic species (ciliates and dinoflagellates; e.g., *Mesodinium* and *Tripos*) and heterotrophs (e.g., heterotrophic dinoflagellates) containing freshly ingested prey (Fig. 3B). Mixotrophs were most abundant in the PFZ (41 ± 15 cells ml^−1^; 0.7 ± 0.2% of all cells counted) where biomass was high and iron concentrations were low (Figs. 2E and 3A and fig. S1), while heterotrophs were most abundant in the SAZ [35 ± 28 cells ml^−1^ (1.3 ± 1.9%)]. The lowest abundances of mixotrophs and heterotrophs were observed in the MIZ [8 ± 4 cells ml^−1^ (0.2 ± 0.04%) and 12 ± 5 cells ml^−1^ (0.3 ± 0.1%), respectively]. Although the two groups were similarly abundant across the transect, the mixotrophs contributed more to biovolume (Fig. 3C) as they were ~2.5 times larger than the heterotrophs (average total biovolume of 436 ± 391 μm^3^ versus 167 ± 102 μm^3^).

The mesozooplankton community, characterized via microscopy, was numerically dominated by copepods (90 ± 8%) at all the experimental stations, with most of the mesozooplankton (copepods and other groups) being mature adults (83 ± 4%; Fig. 3D). We observed the lowest relative abundance of copepods (79%) in the PFZ where satellite chlorophyll a data indicate that the phytoplankton bloom had progressed the furthest by the time of our sampling (surface chlorophyll a concentration of 1.4 μg liter^−1^; Fig. 2F and fig. S1). In the OAZ where the phytoplankton bloom had not progressed as far (surface chlorophyll a of 0.7 μg liter^−1^), copepods dominated the community (97%). Overall, mesozooplankton were most abundant in the MIZ (848 individuals liter^−1^), with high abundances also observed in the PFZ and OAZ (783 individuals liter^−1^ and 669 individuals liter^−1^, respectively), and least abundant in the SAZ (99 individuals liter^−1^).

### Primary production and nutrient uptake rates

**Fig. 4.**
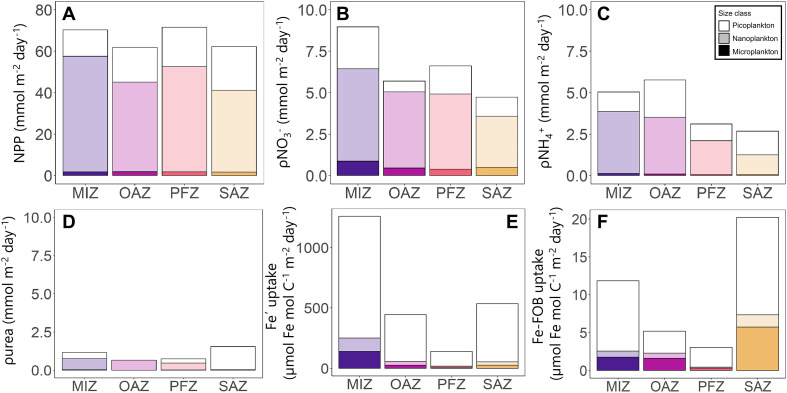
Rates of primary production and nutrient uptake. Bar plots showing the mixed-layer integrated rates of (**A**) net primary production (NPP), (**B**) nitrate uptake (ρNO_3_^−^), (**C**) ammonium uptake (ρNH_4_^+^), (**D**) urea uptake (ρurea), (**E**) dissolved inorganic iron uptake (Fe′ uptake) normalized to POC, and (**F**) organically complexed iron uptake (Fe-FOB uptake) normalized to POC. The shading denotes the different plankton size classes (white, picoplankton; opaque, nanoplankton; solid color, microplankton).

The rates of NPP were similar at all experimental stations (average mixed-layer integrated rate of 66.5 ± 5.2 mmol m^−2^ day^−1^; Fig. 4A, fig. S2A, and table S1), with nanoplankton contributing most to total NPP (73 ± 9%), while the picoplankton contributed 25 ± 9% and the microplankton contribution was low (2.4 ± 0.9%). The highest mixed-layer integrated rate of ρNO_3_^−^ (i.e., new production) was measured in the MIZ (9.0 ± 0.01 mmol m^−2^ day^−1^) and the lowest in the SAZ (4.7 ± 0.01 mmol m^−2^ day^−1^) (Fig. 4B and fig. S2B). As per NPP, the nanoplankton contributed most to total ρNO_3_^−^ at all stations (67 ± 17%), while the average pico- and microplankton contributions were 25 ± 16% and 8 ± 3%.

The rates of regenerated N uptake (i.e., ρNH_4_^+^ and ρurea) were ~1.4 times lower than ρNO_3_^−^ (Fig. 4, C and D, and fig. S2, C and D), with ρNH_4_^+^ accounting for 63 to 93% (average of 80%) of regenerated N uptake across the transect. ρNH_4_^+^ and ρurea generally varied with the ambient NH_4_^+^ and urea concentrations. The highest mixed-layer integrated rates of ρNH_4_^+^ were observed in the OAZ (5.8 ± 0.04 mmol m^−2^ day^−1^) and the lowest in the SAZ (2.7 ± 0.01 mmol m^−2^ day^−1^). As for NPP and ρNO_3_^−^, the nanoplankton contributed most to total ρNH_4_^+^ (63 ± 12%), while the picoplankton contribution averaged 35 ± 12%, reaching a maximum in the SAZ (48 ± 13%) where picoplankton abundances were highest (Fig. 3B). The microplankton contribution was low at all stations (2 ± 1%). In contrast to ρNO_3_^−^ and ρNH_4_^+^, the highest rate of total ρurea was measured in the SAZ (1.5 ± 0.0 mmol m^−2^ day^−1^) (Fig. 4D). Here, the picoplankton dominated ρurea (98 ± 0.7%), with negligible contributions from the other size classes. The lowest rates of ρurea were measured in the OAZ (0.65 ± 0.0 mmol m^−2^ day^−1^), attributable to the nanoplankton (99 ± 0.1% of total ρurea). At the PFZ and MIZ stations, the nanoplankton dominated ρurea (62 ± 2.7%), with the pico- and microplankton contributing 36 ± 3% and 2.0 ± 0.4%, respectively.

The specific rates of carbon fixation (*V*_C_) and N uptake ($V_{{\mathrm{NO}}_{3}^{-}},V_{{\mathrm{NH}}_{4}^{+}},V_{\mathrm{urea}}$) were generally lowest in the SAZ and increased southward for all size classes (figs. S3 and S4). As per the transport rates (NPP, ρNO_3_^−^, ρNH_4_^+^, ρurea), the specific rates of regenerated N uptake ($V_{{\mathrm{NH}}_{4}^{+}}+V_{\mathrm{urea}}$) were lower than $V_{{\mathrm{NO}}_{3}^{-}}$. The highest specific N uptake rates (for all N species) were associated with the picoplankton, while the nano- and microplankton showed intermediate and low specific uptake rates, respectively.

The concentration of labile inorganic iron ([Fe′]) in the euphotic zone depends on the total dissolved iron concentration ([dFe]) and is also strongly affected by light, temperature, and pH (*42*). The Fe′ uptake rates, determined using subsaturating amendments of ^55^Fe, generally tracked the Fe′ concentrations (table S2). Uptake rates were highest at high light, consistent with enhanced photoreductive generation of Fe′ (*42*), and the surface uptake rates increased with decreasing sea surface temperature, consistent with the increased stability of reduced iron at lower seawater temperatures (fig. S2) (*42*). The highest Fe′ uptake rates were measured in the MIZ (1259.2 μmol Fe mol C^−1^ m^−2^ day^−1^) and the lowest in the PFZ (140.5 μmol Fe mol C^−1^ m^−2^ day^−1^) (Fig. 4E). In contrast to NPP and ρNO_3_^−^, Fe′ uptake was dominated by the picoplankton at all stations (61 ± 26%), while the nano- and microplankton contributed 19 ± 17% and 20 ± 12%, respectively.

In seawater, the composition of organically complexed iron is generally unknown (*42*). We amended seawater with the model iron siderophore ferrioxamine-B (Fe-FOB) (1 nM final concentration) to probe saturated uptake rates. Despite this elevated concentration, the Fe-FOB uptake rates were 1 to 2 orders of magnitude lower than the rates of Fe′ uptake (Fig. 4F). The highest Fe-FOB uptake rates were measured in the SAZ (20.1 μmol Fe mol C^−1^ m^−2^ day^−1^), coincident with the highest abundance of picoplankton and the largest biovolume of heterotrophs (Fig. 3, B and C), while the lowest rates occurred in the PFZ (3.0 μmol Fe mol C^−1^ m^−2^ day^−1^). The picoplankton contributed most to total Fe-FOB uptake (51 ± 32%), with the nano- and microplankton contributing 17 ± 10% and 32 ± 23%, respectively.

### Fe:C and Fe:N uptake ratios

**Fig. 5.**
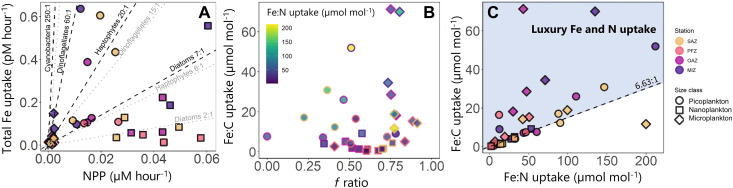
Iron requirements of the different plankton size class. Scatter plots of (**A**) total iron uptake versus NPP, (**B**) the iron-to-carbon (Fe:C) uptake ratio versus the *f* ratio, and (**C**) the Fe:C uptake ratio versus the iron-to-nitrogen (Fe:N) uptake ratio determined for each experimental depth. The symbol shapes in all panels indicate the size classes (circle, picoplankton; square, nanoplankton; diamond, microplankton). The colors in (A) and (C) denote the stations (MIZ, purple; OAZ, pink; PFZ, orange; SAZ, yellow), and those in (B) denote the Fe:N uptake ratios. The black dashed lines in (A) show literature-based average Fe:C uptake ratios (cyanobacteria, 250:1; dinoflagellates, 60:1; haptophytes, 20:1; diatoms, 7:1), and the gray dashed lines show the literature-based minimum Fe:C uptake ratios (cyanobacteria, 200:1; dinoflagellates, 15:1; haptophytes, 6:1; diatoms, 2:1) for the main phytoplankton groups identified in this study (*30*). The black dashed line in (C) shows the Redfield C:N ratio (6.63:1), and the blue shaded area indicates the data points associated with coincident luxury iron and NO_3_^−^ uptake.

Using the size-fractionated rates of NPP and total N (i.e., NO_3_^−^ + NH_4_^+^ + urea) and iron (i.e., Fe′ + Fe-FOB) uptake, we estimated the Fe:C and Fe:N uptake ratios for the three size classes (Eq. 5) (*43*, *44*). At all stations, the nanoplankton were associated with the lowest Fe:C and Fe:N uptake ratios (average of 2.8 ± 0.8 μmol mol^−1^ and 18.8 ± 4.5 μmol mol^−1^, respectively). The microplankton showed the highest Fe:C uptake ratios (average of 26.4 ± 4.1 μmol mol^−1^), while the picoplankton were associated with the highest Fe:N uptake ratios (average of 77.8 ± 16.4 μmol mol^−1^) (Fig. 5, A and C, and table S1).

### Metrics of carbon export potential

**Fig. 6.**
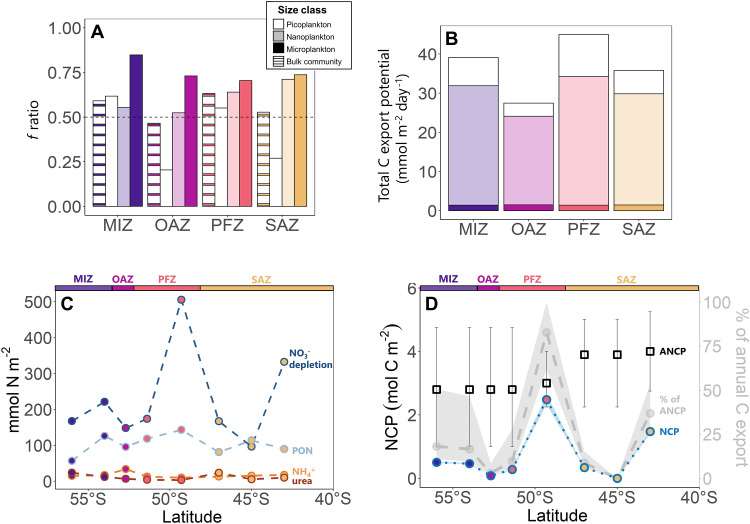
Estimates of carbon export potential and carbon export. Bar plots showing (**A**) the *f* ratios and (**B**) rates of total carbon export potential determined at the experimental stations, with the shading indicating the different plankton size classes (white, picoplankton; opaque, nanoplankton; solid color, microplankton) and the striped bars in (A) showing the bulk community estimates. (**C**) Mixed-layer integrated values of NO_3_^−^ depletion (dark blue dashed line; Eq. 9) and particulate organic nitrogen (PON; light blue dashed line), ammonium (NH_4_^+^; yellow dashed line), and urea concentrations (red dashed line) at the time of our sampling, and (**D**) springtime net community production (NCP) estimated using the NO_3_^−^ depletion data (blue dotted line; Eq. 8; left *y* axis), previously estimated rates of annual NCP (ANCP) from the Atlantic Southern Ocean (*35*) (black squares; with error bars indicating the SD of 5° latitude binned estimates), and the fraction of ANCP (%) that can be attributed to the spring bloom (gray dashed line; right *y* axis), with the propagated error of the estimates indicated by the shading. On all panels, the different zones are indicated by the colors (MIZ, purple; OAZ, pink; PFZ, orange; SAZ, yellow).

We calculated the depth-integrated *f* ratio (i.e., the proportion of total production fueled by NO_3_^−^ uptake) (*6*) at the experimental stations (Eq. 6; Fig. 6A). The bulk community *f* ratio was similar at all stations and averaged 0.55 ± 0.07 (range of 0.47 to 0.63), meaning that 55 ± 7% of the photoautotrophically produced organic carbon (NPP) was potentially exportable. The microplankton were associated with the highest *f* ratio (average of 0.75 ± 0.06; range of 0.70 to 0.85), while the nanoplankton *f* ratio was 0.61 ± 0.08 (range of 0.52 to 0.71) and the picoplankton *f* ratio, which varied most across the transect, averaged 0.41 ± 0.20 (range of 0.20 to 0.62).

To estimate the quantity (rather than fraction) of NPP that was potentially exported, we calculated total carbon export potential (i.e., mixed-layer integrated NPP × *f* ratio; Eq. 7; Fig. 6B), which was highest in the PFZ (45.0 ± 0.2 mmol m^−2^ day^−1^) where the rates of NPP were highest and lowest in the OAZ (27.5 ± 0.1 mmol m^−2^ day^−1^) where NPP was lowest (Figs. 2F, 3A, and 4A). Although the microplankton were associated with the highest *f* ratios, they contributed least to carbon export potential (transect average of 4 ± 1%) because of their minor contribution to biomass and NPP. The nanoplankton contributed most to carbon export potential (transect average of 78 ± 4%) due to their large contribution to biomass and NPP.

We additionally estimated NCP (a direct measure of export) using the change in the mixed-layer NO_3_^−^ pool between the period of maximum winter recharge and our sampling (*35*). By the time of the cruise, mixed-layer NO_3_^−^ had declined by 96.5 to 505.5 mmol m^−2^ (average of 251.9 ± 134 mmol m^−2^; Fig. 6C, dark blue dashed line) relative to that available following winter mixing (Eq. 9). The amount of NO_3_^−^ consumed in the mixed layer by the time of our sampling should equal the N (particulate and dissolved, organic and inorganic) remaining in the mixed layer plus the N that has already been exported via the sinking flux. We estimate that 0 to 348.9 mmol N m^−2^ (125.8 ± 120.3 mmol N m^−2^ on average) was exported between the beginning of the growth season and our sampling. Multiplying this value by a range of phytoplankton C:N ratios (Eq. 9), we calculate that 0 to 2.5 mol C m^−2^ (average of 0.7 ± 0.8 mol C m^−2^; Fig. 6D, blue dashed-dotted line) was exported from the mixed layer by mid-November (~2.5 months since maximum NO_3_^−^ recharge).

## DISCUSSION

It is well known that light and iron [and at times, Si(OH)_4_] exert a strong control on productivity and biological carbon export across the Atlantic sector of the Southern Ocean (*1*, *2*, *31*, *45*). In spring, the shoaling of the mixed layer alleviates light limitation, allowing phytoplankton to consume the macro- and micronutrients supplied during winter, thus initiating a bloom (*33*, *34*). In the open Atlantic Southern Ocean, the spring bloom is generally described as being dominated by a mixed phytoplankton community (diatoms and flagellates) north of the PF and by large, heavily silicified diatoms south of the PF that are well adapted to the low (albeit not limiting) iron conditions and are resistant to zooplankton grazing (*23*–*26*). Our springtime data contradict this characterization. We observed nanoplankton (2.7 to 20 μm; predominantly the diatom, *Chaetoceros* spp.) dominating the phytoplankton biomass and rates of NPP and new production across all hydrographic zones of the Atlantic Southern Ocean in spring. Below, we examine the mechanisms underpinning the success of *Chaetoceros* and explore the implications for carbon export and for the succeeding (i.e., summertime) phytoplankton communities.

### Nanoplankton dominate biomass and productivity across the Atlantic Southern Ocean in spring

Nanoplankton contributed >50% of the total phytoplankton biomass and rates of NPP and N uptake at all our experimental stations in early spring (Figs. 2F, 3A, and 4, A to D). The unequivocal dominance of this size class was unexpected as microplankton (particularly large diatoms) have been reported to dominate Southern Ocean productivity in spring and summer, particularly in the PFZ and AZ (the latter being the combined OAZ and MIZ) (*13*, *14*). Microplankton dominance is consistent with the basic tenets of phytoplankton ecophysiology—phytoplankton resource utilization traits typically depend on cell volume, with nutrient affinity and growth rates declining as cell volume increases (*8*, *46*, *47*). Large cells are thus expected to dominate when nutrients are readily available (i.e., early in the Southern Ocean’s growth season), while small cells should proliferate under nutrient-deplete conditions (e.g., in late summer and autumn) (*48*–*50*). However, across the Atlantic Southern Ocean in spring, microplankton dominated neither biomass nor productivity.

We hypothesize that the low microplankton productivity was the consequence of two physiological constraints: (i) the greater effect of low light availability on large cells (*47*) and (ii) the fact that maximum potential growth rates generally decline with increasing cell size (*8*) (figs. S3 and S4). Across our transect, the mixed layer was always deeper than the *Z*_eu_ (table S1), often by >50 m. As such, the phytoplankton community would have experienced some degree of light limitation. The amount of light absorbed by phytoplankton decreases with increasing cell size (*47*); thus, microplankton are more prone to light limitation than pico- and nanoplankton. Light limitation likely hindered the proliferation of the microplankton, resulting in slower growth and nutrient uptake rates, and relatively low biomass (Figs. 2F and 3A and fig. S4). In addition, microplankton are physiologically unable to grow as rapidly as pico- and nanoplankton because of their larger cell volume (*8*), which would have further impeded their success. By contrast, the intermediate size of the nanoplankton would have allowed them to grow more efficiently given the available resources, outcompeting the microplankton. The nanoplankton also outcompeted the picoplankton, which we attribute to their capacity for high maximum nutrient uptake rates and immediate use of newly consumed nutrients for growth, by which they achieve high growth rates (*8*). As such, the nanoplankton would have been able to rapidly consume the replete nutrients as soon as light limitation was alleviated. The physiological “sweet-spot” occupied by the nanoplankton has been hypothesized to explain their dominance across the global ocean under varying physicochemical conditions (*15*–*18*) and appears to also underpin their success during the early stages of the Southern Ocean spring bloom.

Nanoplankton dominance of biomass and productivity was likely also influenced by their apparently low iron requirement (Fe:C uptake ratio of 2.8 ± 0.8 μmol mol^−1^) relative to that of the pico- and microplankton (17.3 ± 6.8 μmol mol^−1^ and 26.4 ± 4.1 μmol mol^−1^, respectively; Fig. 5A and table S1). Iron availability (as inferred from iron concentration) was variable across the transect, with the highest concentrations measured in the MIZ and the lowest in the OAZ (Fig. 2E). However, the ratio in which iron is supplied to the surface layer relative to NO_3_^−^ is more important than the absolute iron flux, with seawater Fe:NO_3_^−^ ratios less than 10 μmol mol^−1^ thought to limit phytoplankton production (*51*). Across our transect, the mixed-layer seawater Fe:NO_3_^−^ ratios ranged from 3 to 27 μmol mol^−1^, indicating that the supply of iron at some stations (i.e., in the OAZ and MIZ) was insufficient to sustain NO_3_^−^ uptake. Assuming balanced phytoplankton growth (i.e., assigning a C:N uptake ratio of 6.7, 7.5, and 7.2 for the SAZ, PFZ and AZ, respectively, as has been recently inferred for phytoplankton biomass in the Atlantic sector) (*52*), we can assess whether the mixed-layer iron concentrations could sustain the Fe:C uptake ratios of the different phytoplankton size classes. Multiplying our measured seawater Fe:NO_3_^−^ by C:N yields theoretical Fe:C uptake ratios of 0.4 to 4.2 μmol mol^−1^ (Fig. 5A and table S1). This exercise reveals that the mixed-layer iron concentrations were sufficient to sustain nanoplankton growth but not pico- or microplankton productivity, further explaining why the nanoplankton were able to thrive at the onset of the spring bloom.

Despite the overlapping size of the diverse nanoplankton cells present during spring (including nanoflagellates, coccolithophores, cryptophytes, and small diatoms; Fig. 3, B and C), each group has distinctive functional traits, resulting in variable success under different physicochemical conditions (*8*). For example, diatoms generally grow faster than other taxa and thrive under high-nutrient, turbulent conditions (*4*, *8*). By contrast, nanoflagellates are better adapted to lower-nutrient, stable conditions, with some species even capable of mixotrophy (*53*). Mixotrophy allows nanoflagellates to persist throughout phytoplankton blooms as they can alternate between autotrophy and heterotrophy depending on light and nutrient availability (*54*, *55*). The grazers identified during our sampling were mostly microzooplankton and copepods (Fig. 3, C and D), which preferentially graze on nanophytoplankton (*24*). Because microzooplankton and phytoplankton have similar lifespans (days), the former can regulate the biomass of the latter (*56*). By contrast, mesozooplankton (the dominant grazers of microphytoplankton) have a more complex life cycle and a much longer life span (weeks to months), such that large phytoplankton can initially outgrow them (*56*, *57*). One might thus expect the nanophytoplankton biomass in the springtime Southern Ocean to have been regulated by the co-occurring microzooplankton, thereby favoring the proliferation of microphytoplankton. Yet, this is not what we observed. We propose that the diversity of the nanoplankton helped this size class dominate the biomass and rates of NPP and new production, with some nanophytoplankton groups (e.g., nanoflagellates) being more readily grazed upon than others (e.g., small diatoms) (*24*). Future elucidation of the contributions of different nanophytoplankton groups, particularly of the dominant species, will yield further insights into the success of this size class during the Southern Ocean spring bloom.

### The springtime nanoplankton community is dominated by *Chaetoceros* spp.

Phytoplankton biovolume was dominated by diatoms at all stations, with diatom chains contributing 43 ± 22% of the total biovolume across the transect (Fig. 3C). These lightly silicified diatom chains mainly comprised *Chaetoceros* spp., the individual cells of which generally fell into the nanoplankton size class (i.e., <20 μm; Fig. 3E). *Chaetoceros* typically dominates in regions that experience unpredictable but rapid alleviation of macronutrient (e.g., upwelling regions) (*18*, *58*) and/or micronutrient limitation (e.g., in the vicinity of Subantarctic Islands) (*59*), and is known to use a “boom-and-bust” growth strategy (*24*, *26*). This strategy involves a period of accelerated growth (boom) that is followed by a rapid decline in the population due to increased competition for diminishing resources and/or enhanced grazing pressure (bust) (*60*). When light limitation is alleviated, *Chaetoceros* responds rapidly and, due to its relatively small size, is able to outcompete the slower-growing larger phytoplankton for nutrients (*18*, *48*). *Chaetoceros* can also outcompete the smaller phytoplankton because it is better adapted to withstand turbulent conditions and is less palatable to grazers (*24*). Recent studies have suggested that the open Southern Ocean spring bloom is characterized by a period of rapid growth during which phytoplankton outpace their dominant grazers, with the highest growth rates achieved before the peak biomass concentrations are reached (*33*, *34*). The subsequent decline in the phytoplankton population is controlled by grazing pressure, with the period of maximum biomass accumulation also associated with declining growth rates (*34*). Our observations are consistent with this suggestion insofar as the *Chaetoceros* bloom involves a period of rapid growth during which potential grazers are outpaced, followed by a period of decline that we suggest is driven by increased grazing pressure (see the next section). We thus propose that the boom-and-bust behavior of *Chaetoceros* controls the evolution of the Southern Ocean’s spring bloom, with both bottom-up and top-down controls rendering this growth strategy favorable.

We suggest that the *Chaetoceros* boom was facilitated, at least in part, by the seemingly low iron requirement of these diatoms. Since *Chaetoceros* dominated total nanoplankton biovolume, we use the nanoplankton Fe:C uptake ratio as representative of their requirement. The nanoplankton Fe:C uptake ratio was much lower than that of the pico- and microplankton (Fig. 5A and table S1), indicating that *Chaetoceros* is well adapted to the perennially low iron conditions of the open Southern Ocean (*19*, *21*, *38*). Additionally, the nanoplankton Fe:C uptake ratios did not rise as the *f* ratio increased (i.e., as the proportion of NO_3_^−^ uptake relative to total N uptake increased; Fig. 5B). This finding is unexpected as NO_3_^−^ assimilation requires considerably more iron than regenerated N consumption due to the high iron demand of the nitrate and nitrite reductase enzymes (*61*). Moreover, NO_3_^−^ assimilation has been shown to be associated with a Fe:C uptake ratio that is ~1.8 times higher than that of regenerated N consumption (*62*). An increase in the *f* ratio should thus have coincided with a higher iron requirement and, consequently, an increase in the Fe:C uptake ratio.

The invariant nanoplankton Fe:C uptake ratios indicate that *Chaetoceros* was using previously stored iron for NO_3_^−^ assimilation and/or operating at a (low) minimum Fe:C quota. Since vacuole-stored iron is generally used by centric diatoms only when they are iron-limited (*63*, *64*), it is unlikely that the near-constant Fe:C uptake ratios of *Chaetoceros* were due to their use of internally stored iron. The idea that phytoplankton were not strongly iron-limited at the time of our sampling is supported by the observation that microplankton in the AZ were engaging in luxury iron (and NO_3_^−^) uptake (Fig. 5C). Under balanced growth conditions, the Fe:C uptake ratio of phytoplankton should equal their Fe:N uptake ratio × C:N ratio (*52*). However, the AZ microplankton Fe:C uptake ratios were roughly four times higher than expected from the Fe:N uptake ratios (i.e., data falling above the dashed line in Fig. 5C), consistent with luxury iron and NO_3_^−^ uptake. Phytoplankton consumption of iron in excess of their immediate metabolic requirements is only feasible when mixed-layer iron concentrations are nonlimiting. Co-occurring luxury iron and NO_3_^−^ uptake has been observed for several Southern Ocean diatom species under conditions of elevated iron (*65*, *66*); the stored nutrients allow these species to continue growing later in the season when iron (and therefore NO_3_^−^) becomes severely limiting (see the “Implications of the spring Chaetoceros bloom for summertime phytoplankton community composition and carbon export potential across the Atlantic Southern Ocean” section and section S3) (*65*, *66*).

The microplankton community in the AZ was dominated by pennate and centric diatoms (Fig. 3C). It is likely that both groups were engaging in luxury iron and NO_3_^−^ uptake as both have a variety of iron and NO_3_^−^ storage mechanisms (*65*, *66*). If the larger diatoms were consuming excess iron, and given that ambient iron was not exhausted by the time of our sampling, it is unlikely that the nanoplankton-sized diatoms were using stored iron. Instead, we hypothesize that smaller diatoms are extremely well adapted to low iron conditions and have thus reduced their iron requirement, assimilating iron and fixing carbon in a particularly low ratio. Previous studies have shown that Southern Ocean *Chaetoceros* are plastic in their Fe:C uptake ratios, reducing them by up to 60% under low iron conditions akin to those observed here (Fig. 2E) (*67*, *68*). The low measured Fe:C uptake ratios of the nanoplankton thus provide insights into the adaptation of Southern Ocean *Chaetoceros* to their environment. That they apparently do not increase their iron requirement during NO_3_^−^ uptake, even when iron concentrations are relatively high, gives them a competitive advantage over other phytoplankton (including other diatoms) during the spring bloom.

We suggest that nanoplankton-sized *Chaetoceros* spp. occupy a unique ecological niche in the open Southern Ocean, as in other environments (*18*, *58*, *59*). Individual *Chaetoceros* spp. cells have all the advantages inherent to being relatively small, while their ability to form long (>50 μm) spiny chains (Fig. 3E) also endows them with beneficial microplankton characteristics. That they can occupy both size classes further selects for *Chaetoceros* dominance during the spring bloom. The nanoplankton-sized cells were able to grow rapidly under low-light conditions, in contrast to the microplankton (fig. S3). At the same time, chain formation allowed *Chaetoceros* to avoid losses due to grazing and deep vertical mixing events, unlike the picoplankton and non–colony-forming nanoplankton (*69*). We suggest that this size duality helps to explain how *Chaetoceros* came to dominate the biomass, productivity, and NO_3_^−^ uptake rates across the large physicochemical gradients that characterize the Atlantic Southern Ocean in spring.

### Implications of the boom-and-bust lifestyle for carbon export

Using NPP and the *f* ratio estimates, we calculate a mixed-layer integrated total carbon export potential of 35.8 ± 0.09 mmol C m^−2^ day^−1^, 45.0 ± 0.09 mmol C m^−2^ day^−1^, 27.5 ± 0.08 mmol C m^−2^ day^−1^, and 39.1 ± 0.06 mmol C m^−2^ day^−1^ for the SAZ, PFZ, OAZ, and MIZ, respectively (Eq. 6; Fig. 6B). These daily rates are high compared to previous summertime estimates derived similarly for the open Atlantic Southern Ocean (summertime average of 16.9 ± 13.2 mmol C m^−2^ day^−1^) (*41*), indicating that spring is an important season for carbon export.

At the time of our sampling, mixed-layer NO_3_^−^ had decreased relative to that available following winter mixing because of phytoplankton growth (Fig. 6C, blue line; Eq. 9). From this NO_3_^−^ depletion, we estimate that 0.7 ± 0.8 mol C m^−2^ (Fig. 6D, blue dashed-dotted line) had been exported from the mixed layer between the start of the growth season when the ambient NO_3_^−^ concentration was highest and mid-November (*35*). Previous estimates of NCP derived from measurements of seasonal NO_3_^−^ depletion indicate that across the Atlantic Southern Ocean, an average of 2.9 mol C m^−2^ are exported annually and that interannual variability is low (*35*). Comparing our springtime estimates to these annual rates of NCP suggests that 24 ± 29% of the carbon export from the Atlantic Southern Ocean mixed layer occurred during the spring bloom (Fig. 6D, gray shaded area). We hypothesize that this carbon export was driven mainly by *Chaetoceros* spp. as a result of their boom-and-bust lifestyle.

The senescence of *Chaetoceros* blooms can be initiated by bottom-up processes such as iron limitation and/or top-down processes such as viral lysis and grazing by micro- (e.g., ciliates) and mesozooplankton (e.g., copepods and krill) (*24*, *27*). Senescence causes *Chaetoceros* chains to form large aggregates and/or resting spores that rapidly sink out of the upper water column, driving large carbon export events (*27*, *70*). The aftermath of *Chaetoceros* senescence was apparent at the PFZ experimental station where the in situ and satellite chlorophyll a data indicate that the bloom had progressed the furthest (Fig. 2F and fig. S1). Here, the relative contribution of diatom chains to biovolume was lowest (mixed-layer average of 30.0 ± 6.9%, versus 46.6 ± 14.9% at the other stations) and the community had shifted toward flagellates and mixotrophs (i.e., nanoflagellates, dinoflagellates, and mixotrophic ciliates; Fig. 3C). Additionally, the mesozooplankton community comprised proportionally more adults and late-stage juveniles than were present at the other stations (Fig. 3D), likely the result of prolonged food availability associated with a more developed plankton community. Furthermore, low mixed-layer iron concentrations and the lowest iron uptake rates were measured at the PFZ station (Figs. 2E and 4, D and E), and mixed-layer NO_3_^−^ was most strongly depleted (by 506 mmol N m^−2^, versus 216 ± 94 mmol N m^−2^ at the other stations).

The satellite chlorophyll a data show that the spring bloom at the PFZ experimental station was initiated in early October (fig. S1). At the adjacent PFZ station (i.e., where rate experiments were not conducted; Figs. 1 and 2), the bloom had not progressed as far, evidenced by the lower mixed-layer chlorophyll a concentration, deeper mixed layer, and elevated mixed-layer iron. Assuming a similarly high initial mixed-layer iron concentration at the PFZ experimental station, we estimate that *Chaetoceros* depleted the available iron in 3 weeks (i.e., October to the time of sampling). Moreover, we hypothesize that as soon as severe iron limitation set in (i.e., seawater Fe:NO_3_^−^ ≤ 10 μmol mol^−1^, as observed during sampling; Fig. 2E), the abundance of *Chaetoceros* declined and senescence was triggered. This decline is evidenced by the increased contribution of mixotrophs and flagellates at the PFZ experimental station, while the community at the adjacent PFZ station was still strongly dominated by diatoms. The contrast between the two PFZ stations underscores how rapidly *Chaetoceros* can consume the available iron and increase its biomass in response to increased light availability.

Our study shows, in agreement with recent work (*34*), that although spring is not the season of maximum biomass accumulation in the Southern Ocean (fig. S1), it is nonetheless a period of elevated NPP and carbon export. The bloom and subsequent rapid senescence of *Chaetoceros* would have facilitated a large carbon export event (*26*), after which the phytoplankton community likely shifted to one dominated by heavily silicified large diatoms and flagellates (e.g., *Fragilariopsis* and dinoflagellates; with increased dominance of these groups observed at the PFZ experimental station; Fig. 3C). Growth of these phytoplankton groups is favored because of their ability to access iron when ambient iron is depleted—by using intracellularly stored iron (diatoms; section S3), via the consumption of organically bound iron, and/or through phagotrophy (flagellates) (*71*–*73*). These adaptations allow the summertime phytoplankton community to persist for longer than the spring bloom species (months versus weeks) (*74*) such that for the growth season as a whole, the summer bloom contributes most to carbon export (e.g., accounting for 75 ± 28% of ANCP in the Atlantic Southern Ocean; Fig. 6D).

That said, the heavily silicified diatom species that dominate the summer bloom export proportionally less carbon per mole of bSi than *Chaetoceros* (*26*, *27*). These large diatoms are considered bSi rather than carbon exporters, as their prolonged persistence in the surface layer leads to in situ recycling of their cellular contents and, ultimately, to the export of carbon-poor frustules (*26*, *27*). The spring-to-summer shift to flagellates also dampens export potential, as these phytoplankton are readily grazed by micro- and mesozooplankton (*24*), which enhances carbon (and nutrient) recycling in the mixed layer (*11*, *12*). As such, while the total amount of carbon exported following the summer bloom is higher than in spring, the proportion of carbon exported relative to that produced in the surface layer may be lower since the summertime export flux comprises relatively more heavily silicified diatoms and mixed-layer carbon recycling is enhanced (*26*, *27*). In addition, peak export associated with the summer bloom appears to occur a few weeks after the period of maximum productivity (*70*, *75*), in contrast to the dynamics of the *Chaetoceros* bloom, which involves a tight coupling between biomass accumulation and carbon export.

### Implications of the spring *Chaetoceros* bloom for summertime phytoplankton community composition and carbon export potential across the Atlantic Southern Ocean

**Fig. 7.**
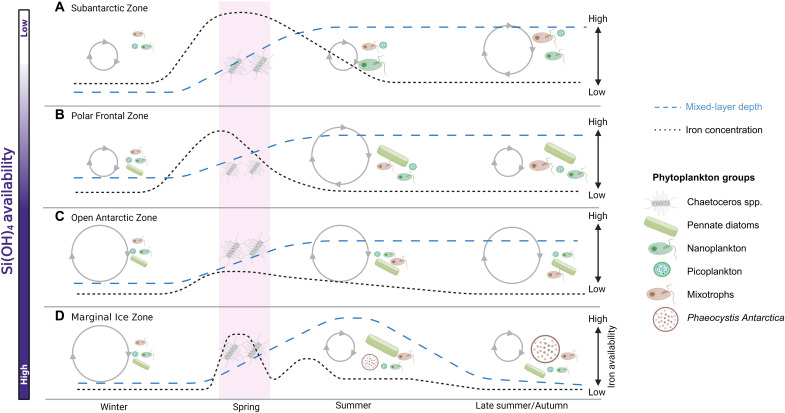
Seasonal evolution of the Southern Ocean phytoplankton community. Schematics showing seasonal physicochemical changes and associated shifts in the phytoplankton community in the (**A**) Subantarctic Zone (SAZ), (**B**) Polar Frontal Zone (PFZ), (**C**) Open Antarctic Zone (OAZ), and (**D**) Marginal Ice Zone (MIZ) of the Atlantic Southern Ocean, developed using our new springtime data and published observations from other seasons (see text for references). The pink shaded region indicates our sampling period. The different phytoplankton groups and their qualitative importance (smaller, less dominant; bigger, more dominant) are shown in each panel. The gradient in mixed-layer silicate [Si(OH)_4_] availability is represented by the bar to the left of the schematic [high (dark purple) to low (white)]. The relative rate (number of arrows) and magnitude (size of circles) of iron and N recycling are shown by the gray circles. The dashed blue line indicates the MLD, and the dotted black line shows the relative iron concentration. Figure created using BioRender.com.

Southern Ocean phytoplankton community composition changes over the growth season in response to synergistic bottom-up (e.g., nutrient and light availability) and top-down (e.g., grazing pressure) processes (*25*, *76*). Below, we place our springtime data into a broader temporal context by synthesizing the existing information available for all seasons, with a particular focus on understanding the implications of the spring bloom for summertime productivity and carbon export (Fig. 7). For all hydrographic zones of the Atlantic Southern Ocean, our observations show that the springtime *Chaetoceros* bloom rapidly consumes mixed-layer iron, likely causing iron limitation of the succeeding summertime community. This limitation should favor phytoplankton groups capable of accessing iron by other means (e.g., stored intracellularly, via the consumption of organically bound iron, and/or through phagotrophy) (*71*–*73*). At the same time, the meridional gradient in Si(OH)_4_ availability [and in the Si(OH)_4_:NO_3_^−^ ratio] drives a divergent pattern in phytoplankton community succession north and south of the PF.

#### Subantarctic Zone

As the growth season progresses, Si(OH)_4_ limitation causes the SAZ phytoplankton community to shift from centric diatoms to flagellates and haptophytes (Fig. 7A) (*66*). This shift facilitates increased iron recycling, as copepod grazing on flagellates and haptophytes (Fig. 3D) results in up to 50% of their iron being immediately released into the mixed layer (*24*, *77*). In addition, the fecal pellets of zooplankton grazing on flagellates and haptophytes sink relatively slowly such that they are dominantly remineralized in the mixed layer, increasing the flux of recycled iron (*32*). Iron still becomes limiting by mid to late-summer (*20*, *21*), however, driving a shift toward smaller cells (including *Synechococcus*) and increased mixotrophy (*29*, *78*). Although small cells have a relatively high iron requirement (Figs. 4, E and F, and 5A) (*30*, *71*), their success is the result of two ecological advantages: (i) their higher surface area–to–volume ratio and (ii) in the case of *Synechococcus* (a dominant picoplankton group in the SAZ; Fig. 3B), the production of siderophores, which allows this taxon to access organically bound iron (Fig. 4F) (*30*, *71*). These factors select for picoplankton over larger cells when iron concentrations become severely limiting (*71*). The coincident increase in mixotrophs is due to this group being able to acquire iron directly via phagotrophy (*72*).

Large spring and early to mid-summer (i.e., November to February) blooms in the SAZ provide organic matter that fuels remineralization in late summer and autumn (i.e., March to May) (*29*). High fluxes of recycled N coincident with low iron favor regenerated production since NH_4_^+^ assimilation requires little iron (*4*, *29*, *41*). In the framework of the new production paradigm, the shift to recycled N uptake decreases the potential for carbon export (i.e., decreases the *f* ratio) (*6*, *29*, *41*). An increase in the proportion of small, slow-sinking phytoplankton will also decrease the direct carbon export flux as these cells are dominantly retained in the mixed layer (*11*, *12*). The shift from *Chaetoceros* to small phytoplankton in the SAZ thus decreases carbon export over the summer. We note that previous summertime studies in the Atlantic sector observed lower rates of carbon export in the SAZ compared to the other hydrographic zones, likely because of strong iron limitation (*20*, *21*).

#### Polar frontal Zone

The phytoplankton community in the PFZ following the spring bloom has been described as the most diverse of the Southern Ocean (*25*, *79*, *80*). The PFZ is a hydrographically dynamic region (*81*), which limits the dominance of one species or group over another (*82*). In spring, the elevated mixed-layer iron and Si(OH)_4_ concentrations may favor luxury iron uptake by large diatoms (*65*, *73*), which, combined with the perennially high Si(OH)_4_ concentrations, allows these diatoms to persist into late summer and autumn (Fig. 7B) (*31*). Coincidently, the PFZ hosts some of the largest spring and summer blooms of the open Southern Ocean (*25*, *83*). The biomass thus produced fuels heterotrophy in late summer, enhancing N recycling in surface waters (*84*). The resultant elevated NH_4_^+^ availability favors the proliferation of small phytoplankton such as nanoflagellates, the grazing of which by zooplankton enhances iron recycling (*77*), as in the SAZ. The rapid recycling of both N and iron allows small phytoplankton to persist throughout the growth season and by its end, the PFZ hosts a mixed community of small phytoplankton, mixotrophs, and heavily silicified diatoms (Fig. 7B) (*25*, *79*, *80*).

This mixed community drives two dominant export regimes: (i) elevated export associated with heavily silicified diatoms (*25*–*27*) and (ii) low export (and *f* ratios) due to enhanced mixed-layer recycling and the proliferation of small phytoplankton (*85*). The PFZ is considered a region of high export, with the composition of the flux controlled by the large, heavily silicified diatoms that dominate surface waters in late summer and autumn (Fig. 7B) (*25*–*27*). These diatoms are inherently more silicified than other diatom species (bSi-to-carbon ratio of ~0.3:1, versus 0.15:1) (*86*), and they consume proportionally more Si(OH)_4_ than they fix carbon under conditions of iron limitation (>>0.3:1) (*26*). Additionally, persisting in the mixed layer results in carbon loss from these cells (*26*). As such, while heavily silicified diatoms contribute disproportionately to the bSi flux, their contribution to carbon relative to bSi export is low, particularly compared to carbon-rich diatom species such as *Chaetoceros* (*25*–*27*). Previous sediment trap studies from the PFZ have generally observed two peaks in the export flux: the first in December following the spring bloom (consistent with our findings), and the second in February/March following the summer bloom, with this export event generally dominated by heavily silicified diatoms (*70*, *75*).

#### Antarctic Zone

The dominance of diatoms in the AZ (*Chaetoceros* in spring and pennate species in summer and autumn) impedes the growth of other phytoplankton groups during periods of iron limitation. Zooplankton grazing on diatoms, particularly heavily silicified species, dampens mixed-layer iron recycling because the bSi frustules remain intact as they pass through the zooplankton gut (*26*). As such, the iron contained in frustules is efficiently exported from the mixed layer in rapidly sinking fecal pellets (*23*), instead of being recycled in the mixed layer. Low rates of mixed-layer iron recycling, in turn, limit the growth of phytoplankton that are unable to store iron or consume organically bound iron (Fig 7, C and D) (*30*).

While there are clear similarities in the phenology of the OAZ and MIZ (as outlined above), differences in the successional trends of their phytoplankton communities have also been observed. The MIZ is strongly influenced by the seasonal cycle of sea ice, in contrast to the OAZ surface waters that remain ice free year round. Sea-ice melt introduces buoyancy that causes a rapid shoaling of the MIZ mixed layer, alleviating phytoplankton from light limitation, which leads to intense blooms in spring (Fig. 2F and fig. S1) (*84*, *87*). Continued sea-ice melt subsequently exposes the MIZ surface waters to enhanced wind-driven mixing that deepens the mixed layer into the late summer, in contrast to the other Southern Ocean zones (*87*). Mixed-layer deepening eventually causes light limitation of phytoplankton, driving a shift toward species adapted to low-light conditions such as *Phaeocystis antarctica* (*88*, *89*), which can occur as either single cells or colonies. The colonies are generally more abundant later in the season as they can store iron within their mucosal matrix, allowing them to persist when iron becomes limiting (*90*). *P. antarctica* can also increase colony size when they experience grazing pressure and are thus less readily consumed than diatoms and other groups (*91*). These adaptations allow *P. antarctica* colonies to remain in the MIZ following the diatom bloom (Fig. 7D) (*88*). The success of *P. antarctica* in the other hydrographic zones is limited, as the mixed layer shoals rather than deepens from summer to early autumn, selecting for alternate phytoplankton groups (*88*, *89*, *92*).

The spring to late-summer shift in the MIZ phytoplankton community from diatoms to *P. antarctica* (*76*, *90*) may enhance carbon export, as *P. antarctica* fix roughly twice as much carbon per mole of phosphate consumed than diatoms, and the colonies have been observed to sink rapidly out of the surface layer (*93*, *94*). However, studies have also shown that following senescence, *P. antarctica* colonies are rapidly degraded in the upper 150 m, thereby up-regulating the microbial loop instead of contributing to carbon removal (*63*, *64*, *89*). The resultant enhanced NH_4_^+^ availability, coincident with low iron, may shift the *P. antarctica–*dominated community toward regenerated N uptake, with the elevated NH_4_^+^ concentrations potentially even inhibiting NO_3_^−^ consumption (*84*, *92*). As such, the shift from diatoms to *P. antarctica* may in net decrease both the *f* ratio and carbon export in the MIZ.

The Atlantic OAZ is generally characterized by low iron conditions (Fig. 2E) because the winter mixed layer is considerably shallower than the ferricline, which limits the amount of iron entrained during winter mixing (*19*, *38*). Productivity is impeded by the low iron concentrations, and phytoplankton growth is predominantly fueled by regenerated N following the spring bloom (*24*). This circumstance also extends to the heavily silicified diatoms that persist throughout the growth season. Although the heavily silicified pennate diatoms have large iron stores (see section S3), they will preferentially consume regenerated N over NO_3_^−^ if the regenerated N flux is sufficient (*95*), a strategy that limits their iron demand and thus enhances their longevity (*95*). The net effect of the shift from *Chaetoceros* to heavily silicified diatom species is thus a decrease in carbon export (and carbon export potential), although the heavily silicified diatoms are still associated with a substantial export flux (*26*, *27*).

Although the evolution of the phytoplankton community following the *Chaetoceros* bloom differs by hydrographic zone, the spring bloom has similar consequences in all zones due to the rapid consumption of iron by *Chaetoceros* (as observed at the PFZ experimental station; Fig. 2E). The phytoplankton communities that proliferate after the spring bloom appear to be adapted to low-iron conditions, continuing to bloom (i.e., the highest biomass and NPP are observed in the summer) and export carbon (*34*, *70*, *75*). Summertime phytoplankton growth accounts for ~75% of ANCP across the Atlantic Southern Ocean (Fig. 6D). However, unlike in spring when productivity and export are tightly coupled, carbon export lags primary production in summer, with a peak in export typically observed weeks after the mid-summer bloom (*70*, *75*). Following this bloom, carbon export potential declines (*41*, *84*, *96*), mainly because iron limitation (and later in the season, reduced light) forces phytoplankton to consume more regenerated N, the supply of which is elevated due to heterotrophic degradation of the spring and summer blooms (*4*, *84*, *92*). This late summer decline in export potential underscores the importance of the spring *Chaetoceros* bloom for Southern Ocean carbon removal on an annual basis.

Over the vast range of physicochemical conditions that characterize the Atlantic Southern Ocean in spring, we observed rates of productivity and carbon export (potential) that were remarkably similar among hydrographic zones. We attribute these trends to the dominance of nanoplankton-sized *Chaetoceros* spp. in all zones, with the boom-and-bust strategy of this taxon allowing it to outcompete all other phytoplankton groups. The *Chaetoceros* bloom is relatively short-lived (a few weeks), with its subsequent senescence accounting for around a quarter of the annual carbon export flux estimated for the Atlantic Southern Ocean (Fig. 6D). These rapid, short-lived blooms thus have large implications for Southern Ocean carbon cycling and also influence the biogeochemical conditions experienced by the succeeding phytoplankton communities. With climate change, the Southern Ocean is predicted to become more stratified (*76*). In currently light-limited regions (e.g., the springtime MIZ), increased stratification will alleviate light limitation and potentially enhance NPP and carbon export (*76*). However, stratification will also decrease the upward iron supply, potentially driving a decline in carbon production and export in perennially iron-limited regions (e.g., the OAZ) (*76*). The unique ecology of *Chaetoceros*, which can exist as both nanoplankton-sized individual cells and microplankton-sized chains, along with its boom-and-bust lifestyle and apparently low iron requirement, may allow this taxon to adapt to a changing climate, perhaps even mitigating the predicted negative effect of climate change on Southern Ocean carbon export (*76*).

## MATERIALS AND METHODS

### Study region and hydrography

Samples were collected aboard the R/V *SA Agulhas II* during the final leg (9 to 13 November 2019) of the Southern oCean seAsonaL Experiment (SCALE) (http://scale.org.za/) spring cruise along the Good Hope line (*39*) in the Atlantic sector of the Southern Ocean. Sampling was conducted at 12 stations, including 4 experimental stations (Fig. 1, colored symbols) and 8 ancillary stations (Fig. 1, gray symbols), spanning the MIZ, the permanently ice-free OAZ, the PFZ, and the SAZ. Hereafter, all stations are referred to by zone. The positions of the hydrographic fronts were determined from temperature and salinity profiles (*40*) measured using a Seabird conductivity-temperature-depth (CTD) profiler. MLD was determined as the depth at which the Brunt-Väisälä frequency squared (*N*^2^; a function of density) reached a maximum. The euphotic zone depth (*Z*_eu_) was determined as the penetration depth of 1% of surface photosynthetically active radiation (PAR). In cases where the PAR sensor was not deployed (*n* = 3 stations), the *Z*_eu_ was determined as follows

$$E_{z}=E_{0}\times e^{-kz}$$ (1)

where *E* is irradiance, *z* is the euphotic zone depth (*Z*_eu_), 0 is the surface, and *k* is the diffuse attenuation coefficient, extracted from satellite data for the sampling period (6 to 15 November 2019; doi:10.5067/ORBVIEW-2/SEAWIFS/L3M/KD/2022).

### Sample collection

Seawater samples were collected during three separate hydrocasts at each station. During the first hydrocast, seawater was collected in 12-liter Teflon-coated GoFlo bottles attached to a powder-coated aluminum frame and CTD with titanium housings following GEOTRACES protocols (*97*). Seawater was decanted from the GoFlo bottles inside a Class 100 clean container laboratory and processed under a laminar flow-hood. During the second and third casts, seawater was collected in 12-liter Niskin bottles, with nutrient samples collected throughout the water column, while samples for chlorophyll a, phytoplankton taxonomy, and the rate experiments were collected from three to six depths in the mixed layer selected based on in situ (down-cast) profiles of temperature, fluorescence, and PAR.

### Nutrient and chlorophyll a concentrations

Nitrate + nitrite (NO_3_^−^ + NO_2_^−^) and silicate [Si(OH)_4_] concentrations were analyzed using a Lachat Quick-Chem flow injection autoanalyzer following standard colorimetric methods (*98*) in a configuration with a detection limit of 0.1 μM and precision for duplicate samples of ≤0.5 μM. The NO_2_^−^ concentrations were measured shipboard by standard colorimetric methods (*98*) using a Thermo Fisher Scientific Genesis 30 Visible spectrophotometer, with a detection limit of 0.05 μM and precision for duplicate samples of ≤0.1 μM. The NO_3_^−^ concentrations were determined by subtracting NO_2_^−^ from NO_3_^−^ + NO_2_^−^.

Ammonium (NH_4_^+^) concentrations were measured shipboard using the fluorometric method (*99*) as described previously (*29*). The detection limit was <0.05 μM, and the precision for duplicate samples was ≤0.1 μM. Urea-N concentrations were measured following the colorimetric method of (*100*) using a Thermo Fisher Scientific Genesis 30 Visible spectrophotometer equipped with a 10-cm path-length cell. The detection limit was 0.05 μM, and the precision for duplicate samples was ≤0.05 μM.

Dissolved iron (dFe) concentrations were measured using a quadrupole inductively coupled plasma mass spectrometer (Agilent 7900) connected to a seaFAST S3 inline preconcentration system (Elemental Scientific) (*101*). The detection limit was 0.02 nM. GEOTRACES standards GSC (2009 GEOTRACES coastal surface seawater), GSP (2009 GEOTRACES Pacific surface seawater), and in-house consensus/reference materials were included in all runs.

For bulk and size-fractionated chlorophyll a concentrations, seawater was filtered through 0.3-μm and 2.7-μm glass fiber (Sterlitech GF-75 and Grade D, respectively) and 20-μm nylon mesh filters (yielding bulk, ≥0.3 μm; picoplankton, 0.3 to 2.7 μm; nanoplankton, 2.7 to 20 μm; and microplankton, ≥20 μm chlorophyll a) that were immediately transferred to 20-ml glass scintillation vials to which 8 ml of 90% acetone was added before the vials were incubated at −20°C for 24 hours (*102*). Extracts were subsequently measured using a Turner Designs Trilogy fluorometer equipped with a chlorophyll a nonacidified module. The detection limit was 0.025 μg liter^−1^.

### Phytoplankton and zooplankton taxonomy

Samples for phytoplankton taxonomy were collected at six mixed-layer depths in 50-ml centrifuge tubes and analyzed in vivo using a pulse-shape recording and imaging flow cytometer (CytoSense, Cytobuoy.com) fitted with a 488-nm laser, fluorescence sensors (yellow/green, 550 nm; orange, 600 to 650 nm; red, 650 to 700 nm), and two scatter sensors for light scattered parallel (forward scatter) and orthogonal (sideward scatter) to the laser beam. This technique yields phytoplankton counts (cell diameters of 1 to 1000 μm) comparable to those obtained with traditional microscopy, although with more reliable counts for cells <5 μm (*103*). It also provides morphological information as the optical profile for each particle is recorded as it travels through the flow cell. Recorded cells were clustered by similarities in optical properties using CytoClus4 (Cytobuoy.com). Cells were assigned to one cluster only, and the same clustering was used for all samples. Cluster identification was supported by the high-resolution microphotographs taken by the instrument (e.g., Fig. 3E). Individual cell volumes were obtained from the total forward scatter signal, which was converted to volume using an empirical conversion formula (*103*), calibrated before the cruise. Biovolumes were obtained as the sum of all cell volumes in each class.

Samples for mesozooplankton taxonomy were collected during the day (MIZ and OAZ stations) and at night (SAZ and PFZ stations) using a Bongo net (200 μm) that was towed vertically from 200 m to the surface at a constant speed of 0.5 m s^−1^. Samples were transferred to 1-liter low-density polyethylene (LDPE) bottles and immediately fixed with buffered formalin (final solution of 4%, v/v). Samples were analyzed ashore using a ZEISS Stemi 508 stereo microscope and ZEISS Primo Star phase-contrast microscope (*104*). Individuals were grouped based on their life stage and broadly categorized into three groups: copepods, euphausiids (krill), and others.

### Rates of NPP and nitrogen and iron uptake

Simulated in situ experiments were conducted to determine the bulk and size-fractionated (i.e., picoplankton, 0.3 to 2.7 μm; nanoplankton, 2.7 to 20 μm; and microplankton, >20 μm) rates of NPP, N uptake (as NO_3_^−^, NH_4_^+^, and urea), and iron uptake [as labile inorganic iron (Fe′) and organically complexed iron (Fe-FOB)]. Seawater was collected from three depths (typically surface, 50 m, and 75 m, with samples for NPP and N uptake collected in Niskin bottles and those for iron uptake collected in GoFlo bottles) and then prescreened through a 200-μm mesh to remove large grazers. All experiments were performed in duplicate. For NPP and N uptake, isotope tracers were added at ~5 to 10% of the assumed ambient substrate concentrations, yielding final concentrations in the bottles of 100 μM NaH^13^CO_3_, 1 μM ^15^N-NO_3_^−^, 0.05 μM ^15^N-NH_4_^+^, and 0.05 μM ^15^N-urea-N; for the rate calculations, tracer enrichments were calculated after cruise using the measured nutrient concentrations. The bottles were incubated on deck for 4 to 6 hours in a custom-built incubator cooled with continuously running surface seawater and equipped with neutral density filters to simulate the relevant light levels. Experiments were terminated via filtration onto 0.3-μm and 2.7-μm combusted (450°C for 8 hours) glass fiber filters (Sterlitech GF-75 and Grade D, respectively) and Milli-Q–rinsed 20-μm nylon mesh. The 20-μm samples were resuspended in 0.2-μm filtered seawater and then refiltered onto combusted 2.7-μm filters. All filters were stored frozen in combusted (500°C for 5 hours) foil envelopes at −80°C pending analysis.

Ashore, filters were oven-dried for 24 hours at 40°C and then folded into tin cups that were analyzed using a Flash 1112 Series elemental analyzer coupled to a Delta V Plus isotope ratio mass spectrometer (EA-IRMS) in a configuration with a detection limit of 2 μg C and 1 μg N. Blanks (combusted unused filters) and laboratory running standards calibrated to International Atomic Energy Agency (IAEA) reference materials were run after every five samples and used to calibrate the sample measurements. Because of the low concentration of particulate organic matter on the 20-μm filters (often below the EA-IRMS detection limit for N), 60 nmol of reagent-grade urea was added to the samples to increase the organic N and C content. Urea was also added to a number of blank filters that were measured to determine δ^15^N and δ^13^C {where δ^15^N, in permil (‰) versus N_2_ in air, = $\left[\frac{{\left({}^{15}N{/}^{14}N\right)}_{\mathrm{sample}}}{{\left({}^{15}N{/}^{14}N\right)}_{\mathrm{standard}}}-1\right]\times 1000$, and δ^13^C, in permil (‰) versus Vienna Pee Dee Belemnite, = $\left[\frac{{\left({}^{13}C{/}^{12}C\right)}_{\mathrm{sample}}}{{\left({}^{13}C{/}^{12}C\right)}_{\mathrm{standard}}}-1\right]\times 1000$} of the added urea (“urea blank”). The measured carbon (POC) and nitrogen (PON) content, δ^15^N and δ^13^C of the 20-μm samples were corrected for the urea blank following

$$\begin{array}{rl} & \delta^{15}N_{\mathrm{corrected}}=\\ & \frac{\left(\delta^{15}N_{\mathrm{sample}}-N\;{\mathrm{content}}_{\mathrm{sample}})-(\delta^{15}N_{\mathrm{urea}\;\mathrm{blank}}-N\;{\mathrm{content}}_{\mathrm{urea}\;\mathrm{blank}}\right)}{N\;{\mathrm{content}}_{\mathrm{sample}}-N\;{\mathrm{content}}_{\mathrm{urea}\;\mathrm{blank}}} \end{array}$$ (2)

$$N\;{\mathrm{content}}_{\mathrm{corrected}}=N\;{\mathrm{content}}_{\mathrm{sample}}-N\;{\mathrm{content}}_{\mathrm{urea}\;\mathrm{blank}}$$ (3)

where δ^15^N can be substituted for δ^13^C and N content can be substituted for C content.

The rates of NPP and N uptake were determined following (*5*) and (*105*) after converting the measured δ^15^N and δ^13^C values to atom % ^15^N and ^13^C. POC, PON, NPP, N and iron uptake (see below) were determined for each filter fraction (i.e., >0.3 μm, >2.7 μm, and >20 μm). Size-fractionated uptake rates were calculated by subtraction (picoplankton, 0.3 to 2.7 μm; nanoplankton, 2.7 to 20 μm; microplankton, 20 to 200 μm), with error propagated according to standard practices.

For the inorganic iron uptake experiments, to maintain near-ambient labile iron concentrations, we precomplexed 0.5 nM ^55^Fe with 10 μM ultraclean EDTA, which allowed us to calculate the labile iron amendment. [Fe′] was computed following (*106*), adjusting for the in situ iron concentration ([Fe]), temperature, pH, and irradiance (table S2). pH was derived from measurements of total alkalinity (TA) and total dissolved inorganic carbon (TC), and the conditional dissociation constants were derived using the values at 10°C and 20°C from (*106*) and extrapolating to colder temperatures as per (*107*). Calculated dark [Fe′] was 3.4 to 6.5 pM, or <1% of ambient dFe, but increased under higher irradiance and lower temperatures. To measure the community uptake rates of organically complexed siderophore iron, we incubated 1 μM ^55^Fe with 1.2 μM desferrioxamine-B (FOB) in lightly acidified (pH = 3) Milli-Q water, before amending the incubation bottles to reach a final concentration of 1 nM ^55^Fe-FOB.

All seawater manipulations were carried out using trace-metal clean procedures and in a particle- and metal-free environment. Seawater was collected in Go-Flo bottles at the same depths as for the NPP and N uptake experiments, transferred to 4-liter acid-cleaned polycarbonate bottles, and amended with either ^55^Fe-EDTA or ^55^Fe-FOB in a metal-free laminar flow hood. Bottles were incubated for 24 hours in a light- and temperature-controlled incubator, with incubations terminated by filtration through 0.2-, 2.7-, and 20-μm polycarbonate filters (Millipore). Filters were rinsed with an oxalate solution to remove adherent (but non-assimilated) iron (*108*), then rinsed with filtered seawater, and stored in a scintillation vial with 10-ml scintillation cocktail.

Ashore, counts per minute were determined using a scintillation counter (TriCarb 2900) and then converted to disintegrations per minute taking into account radioactive decay (specific activity of ^55^Fe is 113 mCi mg^−1^; PerkinElmer) and using a custom quench curve. Iron uptake rates were calculated using the amended plus intrinsic iron concentrations as follows

$$\begin{array}{rl} & d{\left[\mathrm{Fe}\right]}_{\mathrm{uptake}}\{\left(\mu \text{mol Fe mol C}\;{\mathrm{liter}}^{-1}\;{\mathrm{day}}^{-1}\right)=[{\mathrm{Fe}}_{\mathrm{tot}}]\\ & \times \left[\left(\frac{\mathrm{DPM}-\text{filter blank}}{{}^{55}\text{Fe specific activity}}\right)\times {\mathrm{day}}^{-1}\times {\mathrm{vol}}^{-1}\right]\}\times [\mathrm{POC}] \end{array}$$ (4)

where [POC] is the POC concentration in the incubation bottles at the end of the experiments.

Using the size-fractionated rates of NPP, total N (i.e., NO_3_^−^ + NH_4_^+^ + urea) uptake, and total iron (i.e., dFe′ + dFe-FOB) uptake, we estimated the Fe:C and Fe:N uptake ratios (*43*, *44*) for the three plankton size classes as follows

$$\mathrm{Fe}:X\;{\text{uptake ratio}}_{\left(\text{size class}\right)}=\frac{{\mathrm{Fe}}_{\left(\text{size class}\right)}^{\prime }+\mathrm{Fe}-{\mathrm{FOB}}_{\left(\text{size class}\right)}}{X_{\left(\text{size class}\right)}}$$ (5)

where *X* is either NPP or total N uptake and size class refers to pico-, nano-, or microplankton.

### Carbon export potential and NCP

To determine carbon export potential relative to NPP at each station, we calculated the *f* ratio (short hand for flux ratio; a measure of new production relative to total production) using the N uptake rates (*6*)

$$f\;\mathrm{ratio}=\frac{{\rho \mathrm{NO}}_{3}^{-}}{{\rho \mathrm{NO}}_{3}^{-}+{\rho \mathrm{NH}}_{4}^{+}\;+\rho \mathrm{urea}}$$ (6)

Total community carbon export potential (mmol C m^−2^ day^−1^) was then calculated as

Total carbon export potential=fratio×NPP (7)

where NPP was integrated over the mixed layer at each station.

We estimated NCP (a measure of carbon export; mmol C m^−2^) between the start of the growth season and the time of our sampling using the net depletion of mixed-layer NO_3_^−^ from its maximum winter concentration (i.e., following recharge) until our sampling, following the approach of (*35*)

$$\begin{array}{rl} \text{Spring NCP}=\; & \left({{\mathrm{NO}}_{3}}^{-}\;\mathrm{depletion}-[\mathrm{PON}]-[{{\mathrm{NH}}_{4}}^{+}\right]\\ & -[\mathrm{urea}])\times C:N\;\mathrm{ratio} \end{array}$$ (8)

We computed NCP using three different estimates of the C:N ratio—the Redfield C:N ratio of 6.6:1, the C:N ratio estimated by (*52*) from high-resolution float-based measurements for each zone of the Atlantic Southern Ocean, and the bulk biomass C:N ratio that we measured at each station. [PON], [NH_4_^+^], and [urea] are the average measured mixed-layer concentrations of the various species, and NO_3_^−^ depletion was estimated as

$${{\mathrm{NO}}_{3}}^{-}\;\mathrm{depletion}={\left[{{\mathrm{NO}}_{3}}^{-}\right]}_{\mathrm{source}}-{\left[{{\mathrm{NO}}_{3}}^{-}\right]}_{\mathrm{measured}}$$ (9)

where [NO_3_^−^]_source_ is the average NO_3_^−^ concentration directly below the mixed layer at the time of sampling and [NO_3_^−^]_measured_ is the average measured mixed-layer concentration (*84*, *92*). Equation 8 provides a direct measure of export since if NO_3_^−^ supplied during winter mixing is no longer present in the mixed-layer partway into the proceeding growth season (as any form of dissolved inorganic or organic N, or N biomass), then it has to have been exported.

To estimate the fraction of annual NCP (ANCP) accounted for by the spring bloom, we divided our springtime NCP estimates by the average, maximum, and minimum values of ANCP determined by (*35*) for the Atlantic Southern Ocean (Fig. 6D, black squares and error bars). The uncertainty associated with the springtime contribution to ANCP (gray shading in Fig. 6D) was calculated by averaging the values computed using the average, maximum, and minimum ANCP.
