# Supplementary material for: Nanoplankton: The dominant vector for carbon export across the Atlantic Southern Ocean in spring
Source: Sci Adv. 2023 Dec 1;9(48):eadi3059. doi: 10.1126/sciadv.adi3059 (PMC10691778; doi:10.1126/sciadv.adi3059)
Supplement: Supplementary file 1 — Supplementary Text Figs. S1 to S4 Tables S1 and S2 [file sciadv.adi3059_sm.pdf]

Science Advances
AAAS

Supplementary Materials for

Nanoplankton: The dominant vector for carbon export across the Atlantic Southern Ocean in spring

Raquel F. Flynn *et al.*

Corresponding author: Raquel F. Flynn, flyraq001@myuct.ac.za

Sci. Adv. **9**, eadi3059 (2023)
DOI: 10.1126/sciadv.adi3059

This PDF file includes:

Supplementary Text
Figs. S1 to S4
Tables S1 and S2

Supplementary Text

S1. Surface chlorophyll a concentrations over the phytoplankton growth season

Monthly satellite-derived surface chlorophyll *a* data (10.5067/AQUA/MODIS/L3M/CHL/2022) were used to determine when the spring bloom was initiated across the open Atlantic sector of the Southern Ocean. Chlorophyll *a* concentrations were ubiquitously low (<0.2 µg L^{-1}) south of 40°S until early October 2019 when concentrations began to increase, particularly in the Polar Frontal Zone and Marginal Ice Zone. Surface chlorophyll *a* reached a maximum in January/February 2020.

S2. Size-fractionated rates of NPP, and N and Fe uptake

Experiments to determine the size-fractionated rates of net primary production (NPP) and nitrogen (N; as nitrate (NO_3^-), ammonium (NH_4^+), and urea) and iron (Fe; as dissolved inorganic iron (Fe') and organically-complexed iron (Fe-FOB)) uptake were conducted at four stations across the Atlantic Southern Ocean in spring (Fig. 1 and S2). At all stations, NPP and N uptake were dominated by the nanoplankton (2.7-20 µm), while the picoplankton (0.3-2.7 µm) dominated iron uptake. The rates of NPP and N uptake did not vary with depth, in contrast to the iron uptake rates (Fig. S2). The rates of Fe' and Fe-FOB uptake decreased between the surface (55% light level) and the base of the euphotic zone (Z_{eu}; 1% light level), although the decrease was much larger for Fe' uptake. This decrease with depth is likely because Fe(II), the most bioavailable form of iron, is produced by the photochemical reduction of Fe(III), the most thermodynamically stable form of iron in oxic waters (*43*). The availability of Fe(II) is therefore higher in the surface ocean compared to at Z_{eu}, which explains the higher rates of Fe' uptake at the surface (i.e., a light control).

The specific rates of N uptake ($V_{NO_3^-}, V_{NH_4^+}, V_{urea}$) were generally lowest in the SAZ and increased southwards for all size classes (Fig. S3). As per the transport rates (NPP, ρNO_3^-, ρNH_4^+, ρurea), the specific uptake rates of regenerated N ($V_{reg\ N}$; $= V_{NH_4^+} + V_{urea}$) were lower than $V_{NO_3^-}$. In contrast to the transport rates, the highest specific uptake rates of all N species were associated with the picoplankton, while the nano- and microplankton showed intermediate and low specific uptake rates, respectively. In addition, the microplankton experienced a larger decrease in their specific N uptake rates between the surface and Z_{eu}, particularly for $V_{NO_3^-}$, compared to the pico- and nanoplankton (Fig. S4). The larger decrease in the microplankton specific uptake rates with depth relative to the other size classes implicates the greater effect of low light availability on large

cells (*47*). This decrease was more prominent for $V_{NO_3^-}$ due to NO_3^- being more energetically expensive to consume than regenerated N (*3*).

S3. Iron storage mechanisms in centric and pennate diatoms

The summertime phytoplankton community in the Antarctic Zone (OAZ and MIZ) was predominantly composed of large, heavily-silicified diatom species (Fig. 7c-d). These species persist from the spring (Fig. 2c) when they are able to consume iron in excess of their immediate requirements and store it for later use (*64*). Large diatoms such as *Fragilariopsis* and *Thalassiosira* are capable of substantial iron storage (*63, 64, 73*), although the two groups (i.e., pennates and centrics) employ different storage mechanisms. Pennate diatoms store excess iron intracellularly using the protein ferritin (*73*), while centric diatoms appear to store iron in their vacuoles (*63, 64*). The varied iron storage mechanisms result in different cellular lifespans, with pennate diatoms outlasting centrics, in part because ferritin is a more effective iron storage mechanism (*74*). Indeed, it has been suggested that ferritin allows for the storage of >4000 atoms of iron, which can support up to six cell divisions by pennate diatoms growing on NO_3^- (*74*). Using our maximum microplankton growth rate of 0.09 d^{-1} (average V_C determined for microplankton in the OAZ and MIZ), we calculate that it would take ~7.7 days for the microplankton to undergo one cell division (i.e., $\ln(2)/0.09\ d^{-1} = 7.7$ days). Assuming that the pennate diatoms were growing at this rate, then the iron stored in ferritin could have supported their growth for roughly 1.5 months (i.e., 7.7 days per cell division × six doublings = 46 days). We conclude that the iron storage mechanism employed by pennate diatoms explains their dominance over other phytoplankton groups in the late-summer Antarctic Zone (*66*).

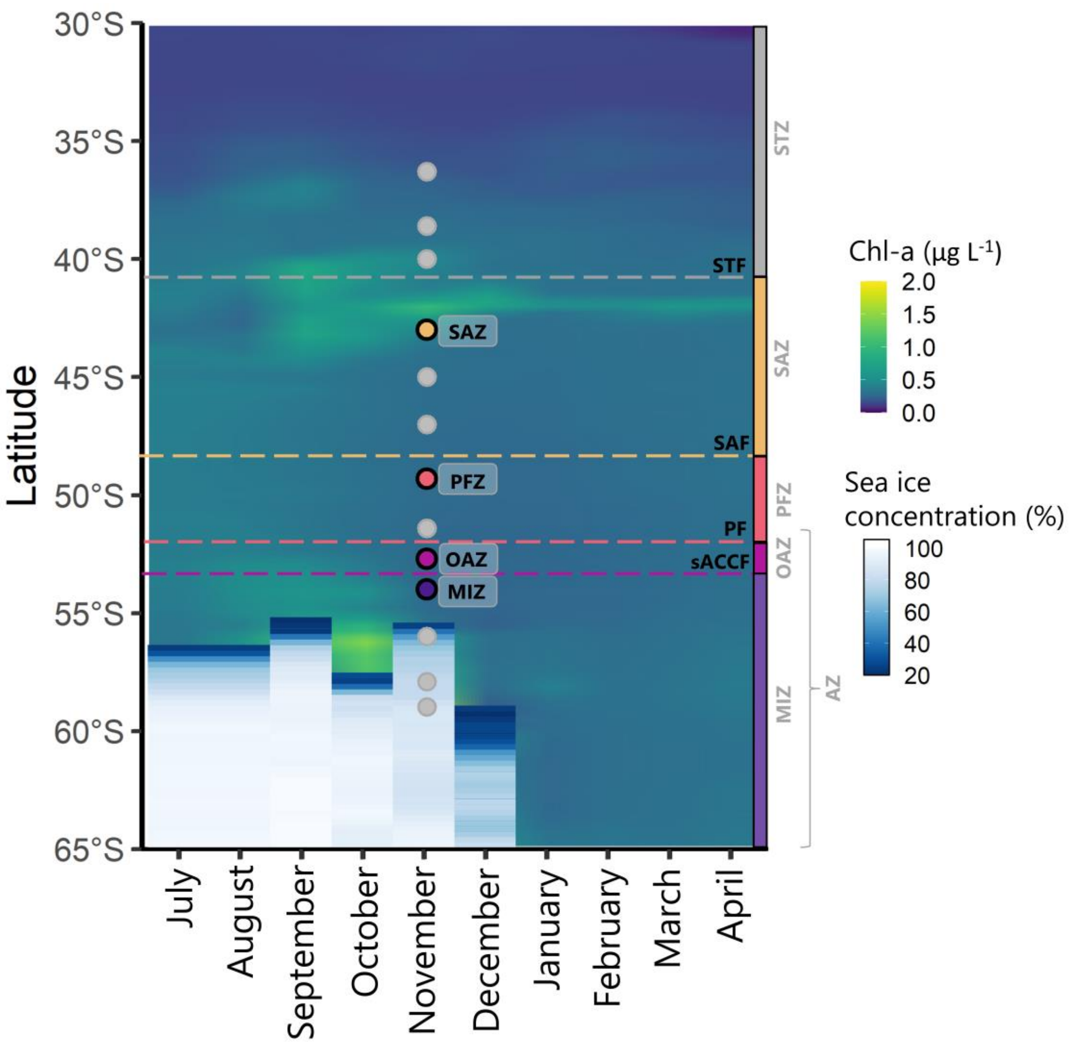

Figure S1. Time series of chlorophyll *a* and sea ice concentrations. Monthly-averaged surface concentrations of chlorophyll *a* and sea ice from July 2019 to April 2020 along the Good Hope Line in the Atlantic Southern Ocean. The stations occupied during the cruise (9-13 November 2019) are denoted by the circles, with the colors indicating the stations where uptake rate experiments were conducted (purple – Marginal Ice Zone (MIZ), pink – Open Antarctic Zone (OAZ), orange – Polar Frontal Zone (PFZ), yellow – Subantarctic Zone (SAZ)) and the grey circles showing the ancillary stations. The positions of the various hydrographic fronts at the time of sampling are denoted by the dashed lines (grey – Subtropical Front (STF), yellow – Subantarctic Front (SAF), orange – Polar Front (PF), and purple – southern boundary of the Antarctic Circumpolar Current Front (sACCF); see main text for details). The chlorophyll *a* and sea ice concentrations are monthly averages from the Copernicus satellite reanalysis product (https://doi.org/10.48670/moi-00019 and https://doi.org/10.48670/mds-00320).

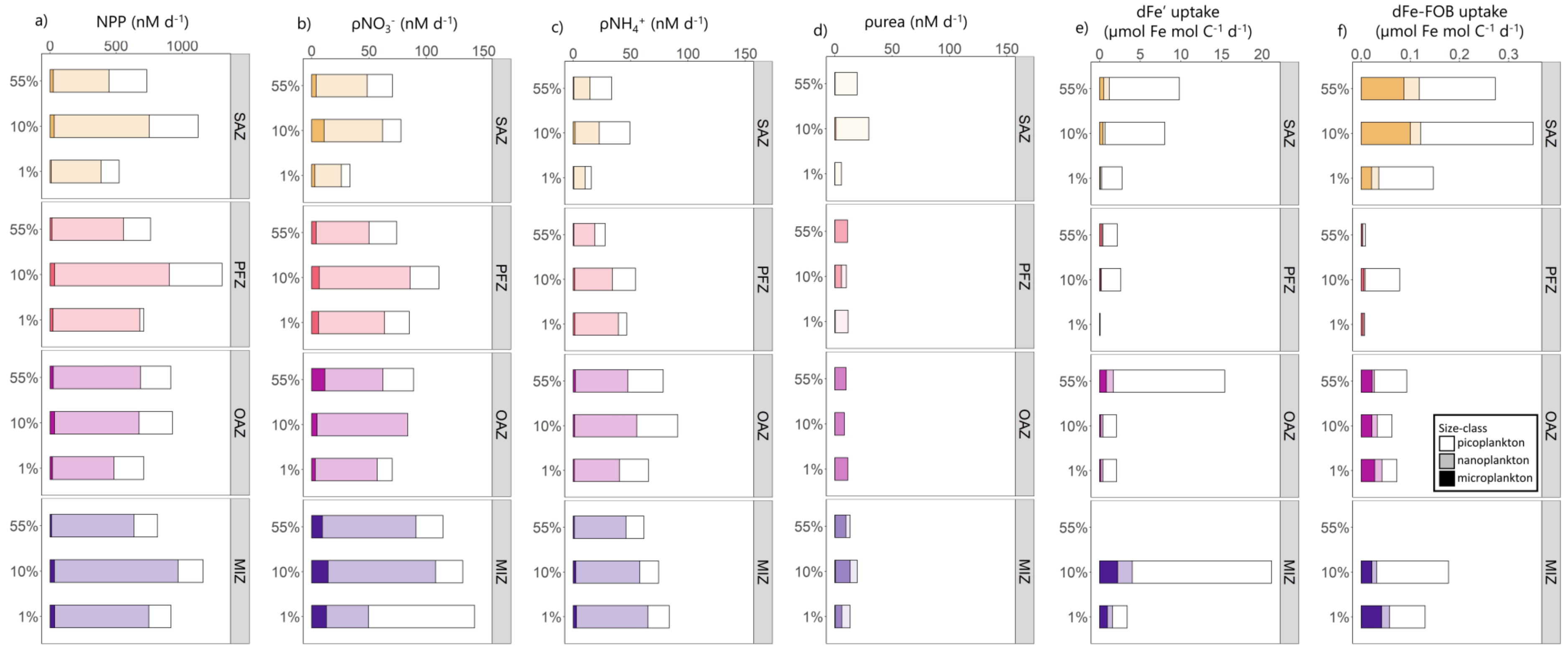

Figure S2. **Rates of primary production and nutrient uptake.** Size-fractionated rates of a) net primary production (NPP), b) nitrate uptake (ρNO_3^-), c) ammonium uptake (ρNH_4^+), d) urea uptake (ρurea), e) dissolved inorganic iron uptake (Fe' uptake), and f) organically-complexed iron uptake (Fe-FOB uptake) in the euphotic zone, shown as a function of light availability relative to surface photosynthetically active radiation (%PAR). The station locations are labelled on the right-hand side of the panels. The colour shading indicates the different plankton size classes (white – picoplankton, opaque – nanoplankton, solid colors – microplankton). Where there are no bars in panels (e-f), no data are available.

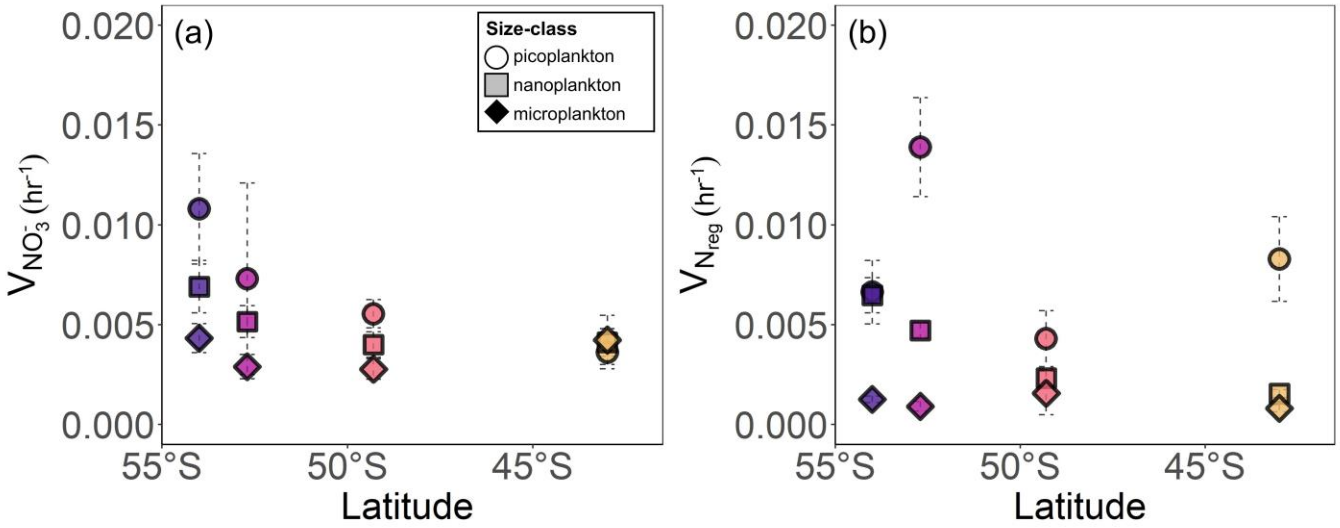

Figure S3. Specific rates of nitrogen uptake. Euphotic zone-averaged specific uptake rates of a) nitrate ($V_{NO_3^-}$) and b) total regenerated nitrogen (i.e., $V_{N\ reg} = V_{NH_4^+} + V_{urea}$) versus latitude at the four experimental stations for the different plankton size classes. The symbols denote size class (circles – picoplankton, squares – nanoplankton, diamonds – microplankton) and the colors indicate station location (purple – Marginal Ice Zone (MIZ), pink – Open Antarctic Zone (OAZ), orange – Polar Frontal Zone (PFZ), yellow – Subantarctic Zone (SAZ)). For all panels, the error bars represent ± 1 SD (n = 6), with error propagated according to standard statistical practices.

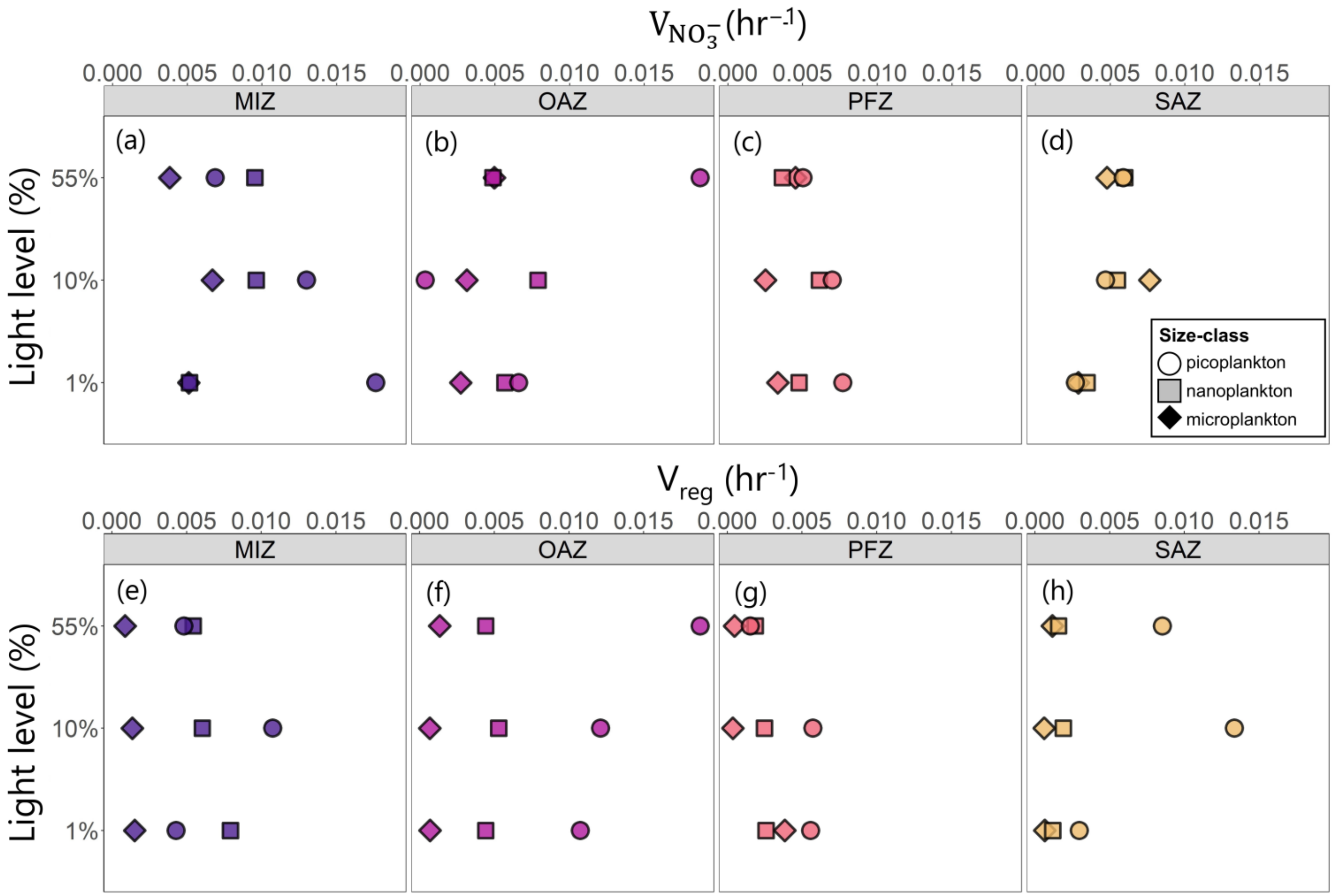

Figure S4. Specific rates of nitrogen uptake. Specific uptake rates of a-d) nitrate ($V_{NO_3^-}$) and e-h) total regenerated nitrogen (i.e., $V_{N\,reg} = V_{NH_4^+} + V_{urea}$) versus light level for all plankton size classes at each station. The symbols denote size class (circles – picoplankton, squares – nanoplankton, diamonds– microplankton) and the colors indicate station location (purple – Marginal Ice Zone (MIZ), pink – Open Antarctic Zone (OAZ), orange – Polar Frontal Zone (PFZ), yellow – Subantarctic Zone (SAZ)).

Table S1. Euphotic zone-integrated rates of net primary production (NPP), nitrate uptake (ρNO_3^-), ammonium uptake (ρNH_4^+), urea uptake (ρurea), dissolved inorganic iron uptake (Fe' uptake), and organically-complexed iron uptake (Fe-FOB uptake), as well as the total iron (i.e., Fe' + Fe-FOB) to carbon (Fe:C) uptake ratio. Values shown are averages ± 1 SD (n = 2 experiments), with error propagated according to standard statistical practices where appropriate. Where standard deviations are not included, the values were <0.01. "-" indicates no available data.

	MLD (m)	Z_{eu} (m)	Size-class	NPP (mmol m^{-2} d^{-1})	ρNO_3^- (mmol m^{-2} d^{-1})	ρNH_4^+ (mmol m^{-2} d^{-1})	ρurea (mmol m^{-2} d^{-1})	dFe' uptake (µmol Fe mol C^{-1} m^{-2} d^{-1})	dFe(FOB) uptake (µmol Fe mol C^{-1} m^{-2} d^{-1})	Fe:C uptake ratios (µmol Fe mol C^{-1})
SAZ	120	39	Bulk	62.2 ± 0.2	4.7 ± 0.01	2.7 ± 0.01	1.5	535.7	20.2	-
			Picoplankton	21.1 ± 0.2	1.2	1.4	1.5	482.5	12.9	20.1
			Nanoplankton	39.5 ± 0.1	3.1	1.2	0.0	27.3	1.6	2.5
			Microplankton	1.7	0.5	0.1	0.0	25.9	5.7	14.9
PFZ	75	37.5	Bulk	71.6 ± 0.1	6.6 ± 0.01	3.1 ± 0.01	0.7	140.5	3.0	-
			Picoplankton	19.0 ± 0.1	1.7	1.0	0.3	122.6	2.6	5.8
			Nanoplankton	50.8 ± 0.08	4.5	2.0	0.4	7.7	0.1	0.7
			Microplankton	1.8	0.4	0.1	0.0	10.3	0.3	7.7
OAZ	150	41	Bulk	61.9 ± 0.2	5.7 ± 0.02	5.8 ± 0.04	0.7	445.5	5.2	-
			Picoplankton	16.8 ± 0.2	0.6	2.3	0.0	387.8	2.9	13.6
			Nanoplankton	43.1 ± 0.07	4.6	3.4	0.6	32.2	0.7	2.8
			Microplankton	1.9 ± 0.02	0.5	0.1	0.0	25.6	1.6	39.4
MIZ	160	42.5	Bulk	70.3 ± 0.05	9.0 ± 0.01	5.0 ± 0.01	1.2	1259.2	11.8	-
			Picoplankton	12.8 ± 0.08	2.5	1.2	0.4	1008.2	9.3	30.5
			Nanoplankton	55.7 ± 0.07	5.6	3.7	0.7	111.5	0.8	6.6
			Microplankton	1.8 ± 0.01	0.9	0.1	0.0	139.5	1.8	52.3

Table S2. Calculated labile iron concentrations ([Fe']) in the experimental bottles given the ambient light, temperature, and pH. pH was derived from measurements of total alkalinity (TA) and total dissolved inorganic carbon (TC). The steady state dissociation constant of the Fe(EDTA) chelate (log k' light) was acquired through linear regression of the data from *(109)* where the temperature-specific dissociation constants are related via log k' = m (pH) + b. Fe[tot] is the sum of the ambient iron concentration and the iron added to the experimental bottles. Values are shown for all experimental depths at each station (listed according to light availability relative to surface photosynthetically active radiation (%PAR)), with the station locations indicated to the left of the Table (Subantarctic Zone (SAZ), Polar Frontal Zone (PFZ), Open Antarctic Zone (OAZ), Marginal Ice Zone (MIZ)).

	% PAR	[Fe'] (pmol L^{-1})	TA (mmol kg^{-1})	TC (mmol kg^{-1})	Temp (°C)	pH	[^{55}Fe] (nmol L^{-1})	$[Fe]_{tot}$ (nmol L^{-1})	[EDTA] (µmol L^{-1})	log k'_{light}	m (°C_{adj})	b (°C_{adj})	Light (µmol m^{-2} s^{-1})
SAZ	55	**12.7**	2262.2	2120.6	6.5	8.021	0.5	0.88	10	-5.985	0.775	12.205	70
	10	**5.6**	2263.3	2120.7	6.5	8.032	0.5	0.88	10	-5.977	0.775	12.205	30
	1	**3.8**	2262.2	2120.6	6.5	8.022	0.5	0.88	10	-5.985	0.775	12.205	21
	DARK	**3.7**	2262.2	2120.6	6.5	8.022	0.5	0.88	10	-7.372	1.241	17.330	DARK
PFZ	55	**14.9**	2272.9	2137.3	3.4	8.055	0.5	0.82	10	-5.886	0.690	11.445	70
	10	**6.4**	2274.7	2137.9	3.4	8.055	0.5	0.82	10	-5.886	0.690	11.445	30
	1	**4.5**	2278.0	2141.0	3.4	8.055	0.5	0.82	10	-5.886	0.690	11.445	21
	DARK	**3.8**	2278.0	2141.0	3.4	8.055	0.5	0.82	10	-7.334	1.103	16.217	DARK
OAZ	55	**17.4**	2295.6	2168.7	0.8	8.043	0.5	0.76	10	-5.787	0.567	10.347	70
	10	**7.4**	2293.5	2171.0	0.8	8.042	0.5	0.76	10	-5.788	0.567	10.347	30
	1	**5.2**	2294.7	2169.9	0.8	8.043	0.5	0.76	10	-5.787	0.567	10.347	21
	DARK	**3.4**	2294.7	2169.9	0.8	8.043	0.5	0.76	10	-7.348	0.903	14.608	DARK
MIZ	10	**13.6**	2292.1	2172.4	-0.3	8.050	0.5	1.25	10	-5.742	0.520	9.925	30
	1	**9.5**	2293.2	2173.9	-0.3	8.046	0.5	1.25	10	-5.744	0.520	9.925	21
	DARK	**5.6**	2293.2	2173.9	-0.3	8.046	0.5	1.25	10	-7.346	0.826	13.989	DARK
